# Past, Present, and Future of Plant-Derived Extracellular Vesicles in Biomedical Applications

**DOI:** 10.3390/ph19081156

**Published:** 2026-07-24

**Authors:** Yilixiati Wusiman, Xiaoxiao Qiu, Nazhakaiti Yusufujiang, Yipaerguli Paerhati, Alifeiye Aikebaier, Dilihuma Dilimulati, Alhar Baishan, Wenting Zhou

**Affiliations:** 1 Department of Pharmacology, College of Pharmacy, Xinjiang Medical University, Urumqi 830017, China; yilixiati@stu.xjmu.edu.cn (Y.W.); qiuxiaoxiao@stu.xjmu.edu.cn (X.Q.); nzkt0110@stu.xjmu.edu.cn (N.Y.); yipaerguli@stu.xjmu.edu.cn (Y.P.); alifeiye@stu.xjmu.edu.cn (A.A.); dilihuma@stu.xjmu.edu.cn (D.D.); alhar@stu.xjmu.edu.cn (A.B.); 2 Xinjiang Key Laboratory of Natural Medicines Active Components and Drug Release Technology, Urumqi 830017, China; 3 Xinjiang Key Laboratory of Biopharmaceuticals and Medical Devices, Urumqi 830017, China; 4 Engineering Research Center of Xinjiang and Central Asian Medicine Resources, Ministry of Education, Urumqi 830017, China

**Keywords:** plant-derived extracellular vesicles, bibliometric analysis, drug delivery, cross-kingdom regulation, gut microbiota, ferroptosis, multi-omics

## Abstract

Plant-derived extracellular vesicles (PDEVs) have emerged as promising natural nanocarriers for biomedical applications owing to their distinctive ability to facilitate intercellular communication and transport bioactive molecules. In this review, we employ bibliometric analysis to identify research hotspots and trends, providing a comprehensive overview of these core themes. The bibliometric results reveal a sustained increase in annual publications in this field, with keyword analysis identifying drug delivery, cross-kingdom regulation, immunomodulation, engineering modification, and gut microbiota as five major research themes. The focus of research has evolved from early basic biological characteristics into engineered smart delivery platforms, with the application areas expanding from intestinal inflammation to neurological, metabolic, dermatological, and oncological diseases. This review systematically examines the core directions in this field. It compares the strengths and limitations of mainstream isolation methods and highlights the value of multi-omics integration, covering the molecular mechanisms of ferroptosis and gut microbiota regulation by PDEVs along with engineering strategies such as drug loading, surface modification, and membrane fusion. It also discusses the latest progress in frontier therapeutic applications of PDEVs, including cancer, inflammatory diseases, tissue regeneration and aesthetics, and neurological disorders. Finally, this review summarizes the key challenges confronting the field, including the lack of standardized protocols, production bottlenecks, and engineering obstacles. It also delineates future directions, including establishing international standardization definitions, advancing multi-omics and AI-driven mechanistic elucidation, developing scalable and efficient purification technologies, and executing systematic preclinical safety and pharmacokinetic evaluations to facilitate clinical translation.

## 1. Introduction

Intercellular communication serves as a central regulatory mechanism governing development, physiological homeostasis, and disease progression in multicellular organisms [1,2]. Extracellular vesicles (EVs) are essential multifunctional carriers that facilitate intercellular communication [3]. In the 1980s, scientists first observed tiny vesicles released by cells in sheep reticulocytes. Subsequently, in 1987, they were formally named “exosomes” [4,5,6,7,8]. In 1996, G. Raposo discovered that B-lymphocyte-secreted exosomes can stimulate anti-tumor immune responses [9]. This discovery established exosomes as a key mediator of intercellular communication, significantly propelling the rapid advancement of EVs research. Exosomes, measuring 30–150 nm in diameter, are secreted via fusion of intracellular multivesicular bodies (MVBs) with the plasma membrane. Nearly all eukaryotic cells are capable of secreting exosomes [10]. Exosomes, abundant in proteins, lipids, and nucleic acids, mediate cellular communication by transferring their contents to recipient cells [11,12,13]. The biocompatibility, low immunogenicity, and transmembrane capabilities of exosomes have demonstrated tremendous application potential in fields such as oncology, gene therapy, and regenerative medicine [14]. However, exosomes derived from animal cells face multiple challenges on the path to clinical application, including production costs, safety, and ethical considerations [15]. Notably, while the modern exosome field originated in the 1980s, the morphological basis for plant-derived extracellular vesicles (PDEVs) was established earlier with Jensen’s observation of MVBs in plant cells in 1965 [16]. PDEVs have garnered considerable interest from researchers because of their distinct benefits, including widespread availability, cost-effectiveness, high biocompatibility, and minimal immunogenicity [17]. PDEVs are nanoscale membrane vesicles (30–400 nm) released from plant cells into the extracellular space through mechanisms such as fusion with the plasma membrane via MVBs or exocytosis [18,19]. Compared with exosomes from animal sources, PDEVs exhibit a phospholipid bilayer structure analogous to that of animal exosomes, while harboring unique bioactive components. These encompass a distinct lipid composition, diverse proteins and nucleic acids, along with numerous plant-specific metabolites [20,21]. PDEVs have attracted growing academic attention in biomedical research, with many review articles published in top journals. While these existing studies provide detailed insights into specific diseases or technologies, a comprehensive, data-driven perspective of the entire subfield is still lacking. To address this gap, we present the first integrated bibliometric analysis of PDEVs in biomedical applications, using literature from PubMed, Web of Science Core Collection (WOSCC), and Scopus. Through quantitative and visual methods, this study delineates the field’s knowledge structure, including its publication trends, research hotspots, and evolutionary frontiers. More importantly, moving beyond pure data mapping, the core research themes and frontiers identified by this analysis are subjected to a detailed review. Given the prevailing terminological ambiguity in this field, we adopt PDEVs as the standard nomenclature for this review.

## 2. Methods

### 2.1. Data Source and Search Strategy

To ensure comprehensive literature coverage and mitigate selection bias, this study performed bibliometric visualization analysis using data integrated from the WoSCC, PubMed, and Scopus databases. As the gold standard for bibliometric studies, WoSCC ensures high data quality and compatibility with analytical software. Scopus compensates with its broad disciplinary breadth, while PubMed provides specialized depth in biomedicine.

Our search strategy employed advanced retrieval based on the Topic and Title fields. In formulating search phrases, we expanded terminology using MeSH terms and rigorously selected keywords and used Boolean operators along with wildcard symbols to optimize both retrieval completeness and result accuracy. Search terms were categorized into three major groups: (1) extracellular vesicle-related terms (“Exosome*”, “Extracellular Vesicle*”, “Nanoparticle*”, “Exosome-like*”, etc.); (2) plant-related terms (“Plant*”, “Herb*”, “Vegetable*”, “Fruit*”, “Botanical”, etc.); (3) specific plant and plant part-related terms (“Ginger*”, “Grape*”, “Broccoli*”, “Root*”, “Leaf*”, “Seed*”, etc.). Due to the lack of standardized nomenclature in PDEVs research, the resulting retrieval formulas were complex and lengthy. To maintain the readability of the text while ensuring complete transparency for reproducibility, the detailed content of the retrieval formulas was provided in Supplementary Table S1.

### 2.2. Inclusion and Exclusion Criteria

Inclusion criteria for this study: (1) Research subjects: EVs isolated from plants. (2) Research content: Involving the isolation, purification, characterization, biological functions, or potential applications of PDEVs in biomedicine. (3) Publication types: Original research articles and review articles. (4) Language: English. (5) Publication date: 1 January 2001–21 May 2026.

Literature exclusion criteria: (1) Irrelevant research subjects: Studies on EVs from non-plant sources. (2) Inappropriate research content: Studies on PDEVs that do not address a biomedical research question, mechanism, or application. (3) Document type: Non-original research articles or review articles. (4) Language: works written in languages other than English.

### 2.3. Study Selection and Database Merge

After initial screening in the databases, the remaining records were manually screened based on titles and abstracts. To minimize selection bias, two authors conducted the screening. Any disagreements were resolved by a third senior researcher. Prior to formal screening, 100 randomly selected records were subjected to double-blind evaluation, and inter-rater reliability was measured using Cohen’s Kappa coefficient [22]. The calculated Kappa coefficient was 0.79, indicating strong consistency between the two evaluators.

**Figure 1:**
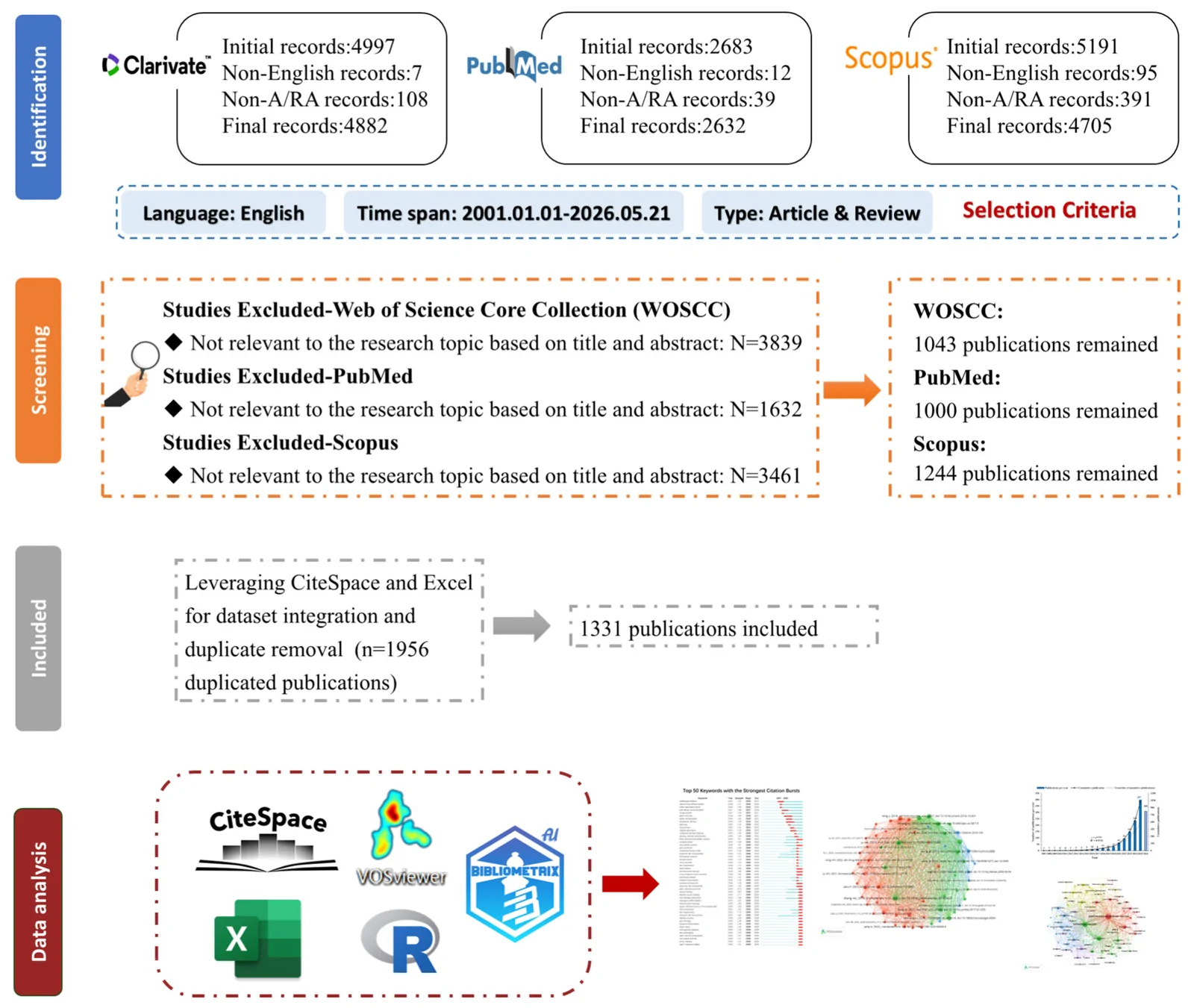
Flowchart of the literature screening process.

A total of 4997 records were retrieved from the WoSCC database. Based on the exclusion criteria, 3954 records were excluded, and ultimately 1043 records that met the inclusion criteria were exported in plain text format as the data foundation for bibliometric analysis. Subsequently, of 2632 records retrieved from PubMed, 1632 were excluded based on the exclusion criteria. De-duplication of the remaining 1000 records was performed in Excel by matching Digital Object Identifier (DOI) against the 1043 eligible articles previously identified from WoSCC. After removing 857 overlapping records, 143 unique records were exported in NBIB format. Finally, 5191 records were retrieved from Scopus and 4705 were exported as CSV files after initial screening. These records were de-duplicated in Excel by matching DOIs against the eligible articles from WoSCC and PubMed. This process removed 1099 overlapping records. Of the remaining 3606 records, 3461 were excluded based on the exclusion criteria, yielding 145 eligible articles that were exported in CSV format. The workflow diagram is illustrated in Figure 1.

Given the superior compatibility of the WoSCC data format, records from PubMed and Scopus were converted to the WoSCC plain text format via CiteSpace to ensure consistency and analytical compatibility with visualization tools.

### 2.4. Data Analysis and Visualization

Microsoft Excel was used for data analysis and chart creation. CiteSpace (6.4.R1) and VOSviewer (1.6.20) were employed for burst detection [23,24] and co-citation network analysis [25], respectively. Quantitative data were extracted using the Bibliometrix R Package (5.0; https://www.bibliometrix.org) (accessed on 25 May 2026) [26]. Furthermore, Gephi (0.10.1) was utilized to calculate network topology metrics to validate the robustness of the clustering structure.

## 3. Results

### 3.1. Trend Analysis of Annual Publications

We included a total of 1331 publications, comprising 1001 original research articles and 330 review papers. Out of these, 15 were written by a single author. These 15 papers were contributed by 14 unique authors, as one author published two single-authored works. The average number of co-authors per publication was 8, and 15.23% of publications involved international collaboration. Furthermore, each paper was cited an average of 23.22 times, reflecting substantial academic impact (Table 1).

**Figure 2:**
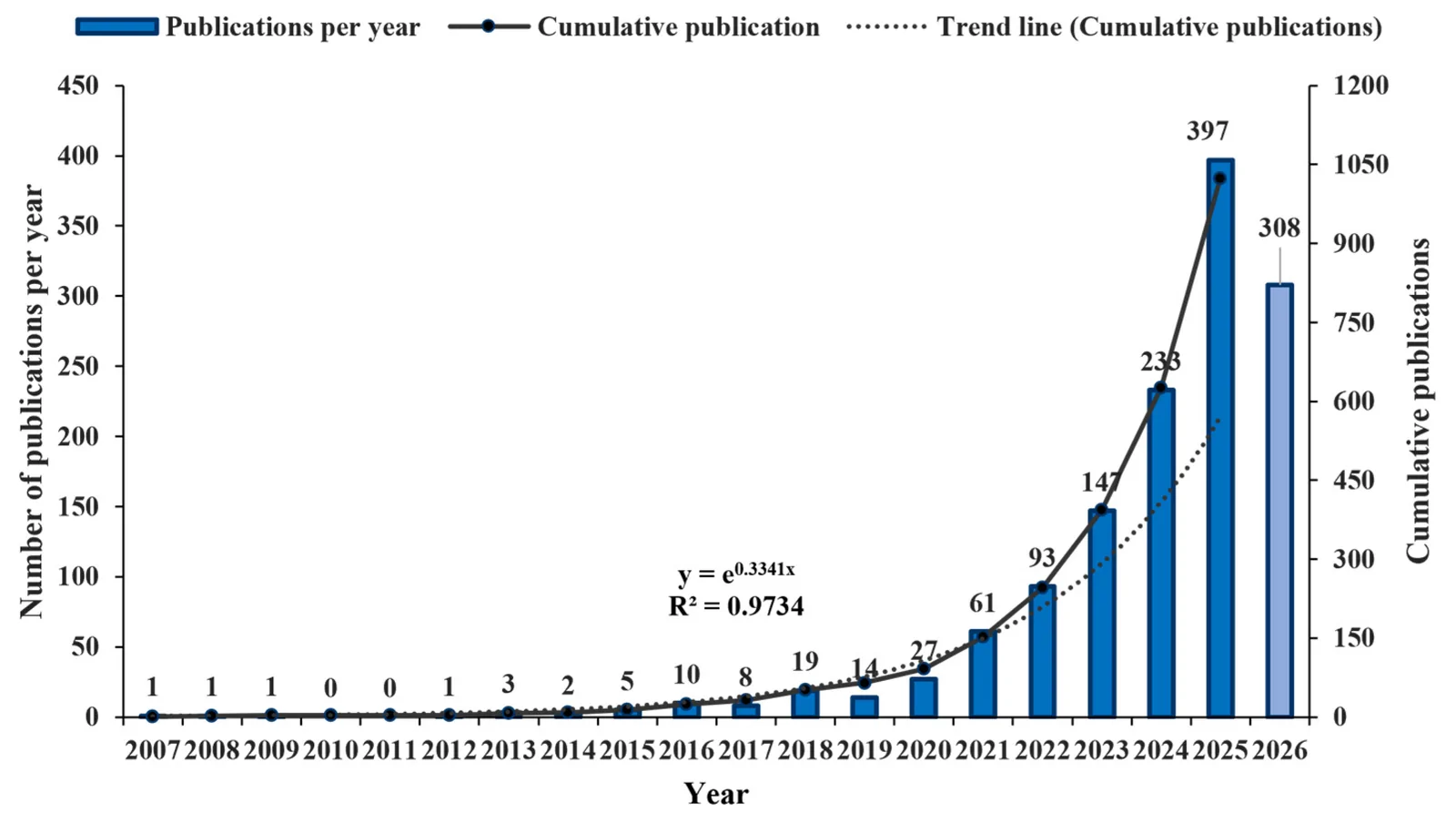
Annual and cumulative number of publications. Data for 2026 are incomplete and cover only January through May.

Figure 2 presents a line chart showing the trend in the number of publications on PDEVs from 2007 to May 2026. The field remained in a nascent phase with minimal output from 2007 to 2015. A turning point occurred around 2017, after which the publication volume surged dramatically, rising from merely 8 publications in 2017 to a peak of 397 in 2025. To quantify this trajectory, we fitted an exponential growth model (y = e^0.3341x^) to the cumulative data spanning 2007 to 2025. The model yielded a strong fit (R^2^ = 0.9734), indicating an estimated average annual growth rate of approximately 39.7%.

This historical growth pattern is corroborated by the 2026 data, covering January to May only, which totaled 308 publications. Although representing less than half the year, this figure already accounts for 78% of the 2025 total output, suggesting the field is maintaining its high growth rate rather than decelerating.

### 3.2. Analysis of Highly Cited and Co-Cited References

The 10 most cited papers on PDEVs are shown in Table 2, eight of which have garnered over 500 citations each. The leading study, by Qiang Cai et al. (2018), has received 1034 citations. Following this, the paper by Ming Zhen Zhang et al. (2017), appearing in *Biomaterials*, has garnered 918 citations, while the study by Yun Teng et al. (2018), published in *Cell Host & Microbe*, has received 907 citations.

**Figure 3:**
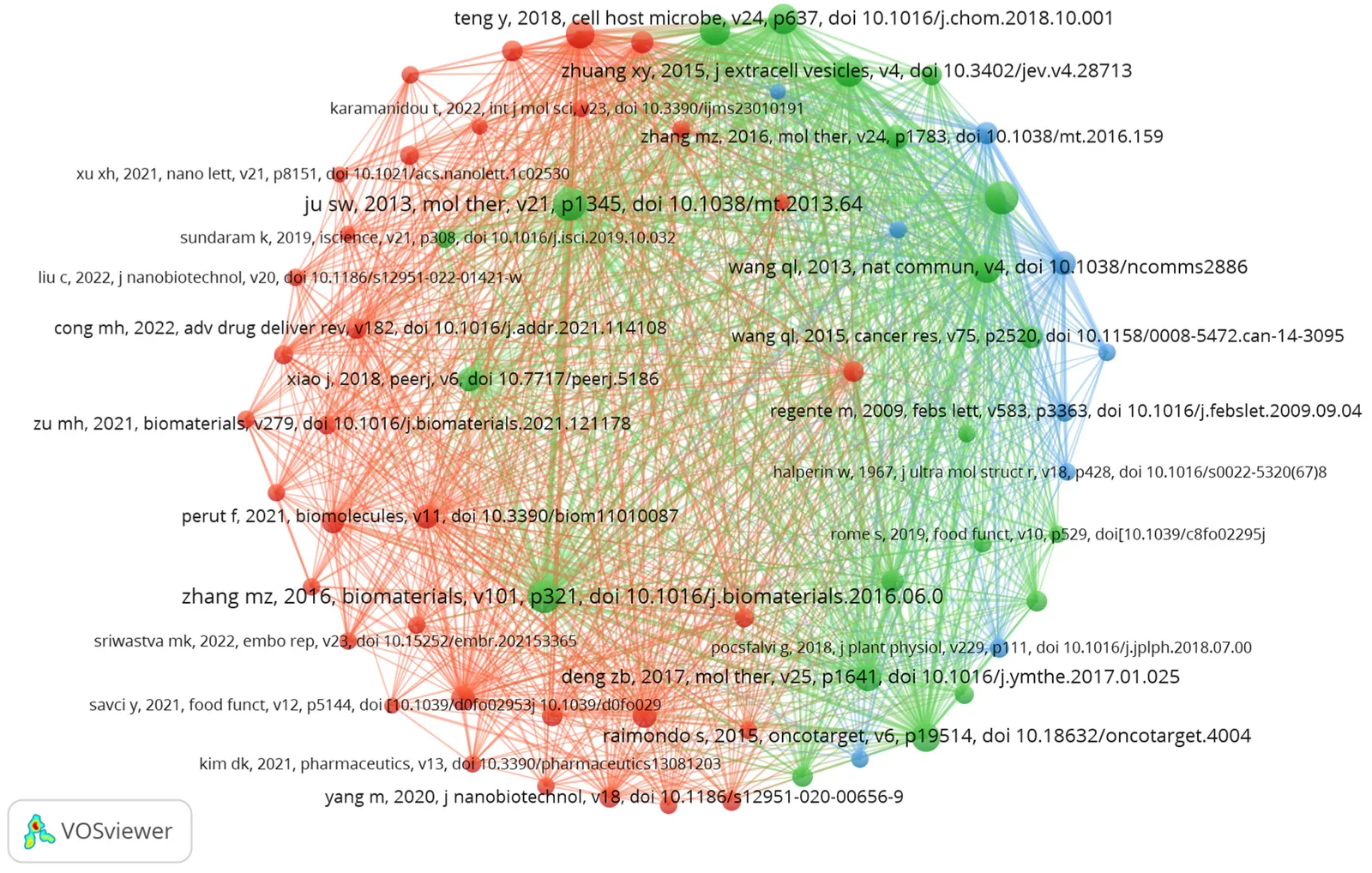
Co-citation analysis of the cited references. In this visualization, each node represents a frequently cited reference, with its size corresponding to total link strength. Nodes are color-coded based on the clustering algorithm, grouping references that are frequently co-cited together into distinct thematic clusters. The links between nodes indicate co-citation relationships, and their thickness represents the strength of that connection.

We employed VOSviewer to perform a co-citation analysis of the cited references, with the resulting network comprising 64 highly cited references (citations ≥ 100; Figure 3). The majority of the top 10 references ranked by total link strength (TLS) were published in *Molecular Therapy* and the *Journal of Extracellular Vesicles* between 2013 and 2018. This strongly underscores the application of PDEVs in molecular therapy as the field’s primary, long-term research focus.

Among the 64 frequently cited references, the studies by Ju et al. (2013) [27], Zhang et al. (2016) [28], and Mu et al. (2014) [29] achieved the highest TLS values in the network and garnered substantial citations. These three works belong to the same co-citation cluster and share exceptionally strong mutual links, confirming their status as a tightly integrated knowledge triad and serving as cornerstones for the field. Details are provided in Supplementary Table S2.

**Table 2:** Top 10 most highly cited literature in the field of PDEVs.

Rank	First Author	Title	Year	Source	Citations
1	Qiang Cai [30]	Plants send small RNAs in extracellular vesicles to fungal pathogen to silence virulence genes	2018	*Science*	1034
2	Mingzhen Zhang [28]	Edible Ginger-Derived Nanoparticles: A Novel Therapeutic Approach for the Prevention and Treatment of Inflammatory Bowel Disease and Colitis Associated Cancer	2016	*Biomaterials*	918
3	Yun Teng [31]	Plant-Derived Exosomal MicroRNAs Shape the Gut Microbiota	2018	*Cell Host & Microbe*	907
4	Songwen Ju [27]	Grape Exosome-like Nanoparticles Induce Intestinal Stem Cells and Protect Mice From DSS-Induced Colitis	2013	*Molecular Therapy*	799
5	Jingyao Mu [29]	Interspecies communication between plant and mouse gut host cells through edible plant derived exosome like nanoparticle	2014	*Molecular Nutrition & Food Research*	646
6	Haseeb Anwar Dad [32]	Plant Exosome-like Nanovesicles: Emerging Therapeutics and Drug Delivery Nanoplatforms	2020	*Molecular Therapy*	625
7	Meng Cao [33]	Ginseng-derived nanoparticles alter macrophage polarization to inhibit melanoma growth	2019	*Journal for ImmunoTherapy of Cancer*	506
8	Baomei Wang [34]	Targeted drug delivery to intestinal macrophages by bioactive nanovesicles released from grapefruit	2013	*Molecular Therapy*	555
9	Qilong Wang [35]	Delivery of therapeutic agents by nanoparticles made of grapefruit-derived lipids	2013	*Nature Communications*	497
10	Stefania Raimondo [36]	Citrus limon-derived nanovesicles inhibit cancer cell proliferation and suppress CML xenograft growth by inducing TRAIL-mediated cell death	2015	*Oncotarget*	487

### 3.3. Keywords’ Co-Occurrence and Burst Detection Analysis

**Figure 4:**
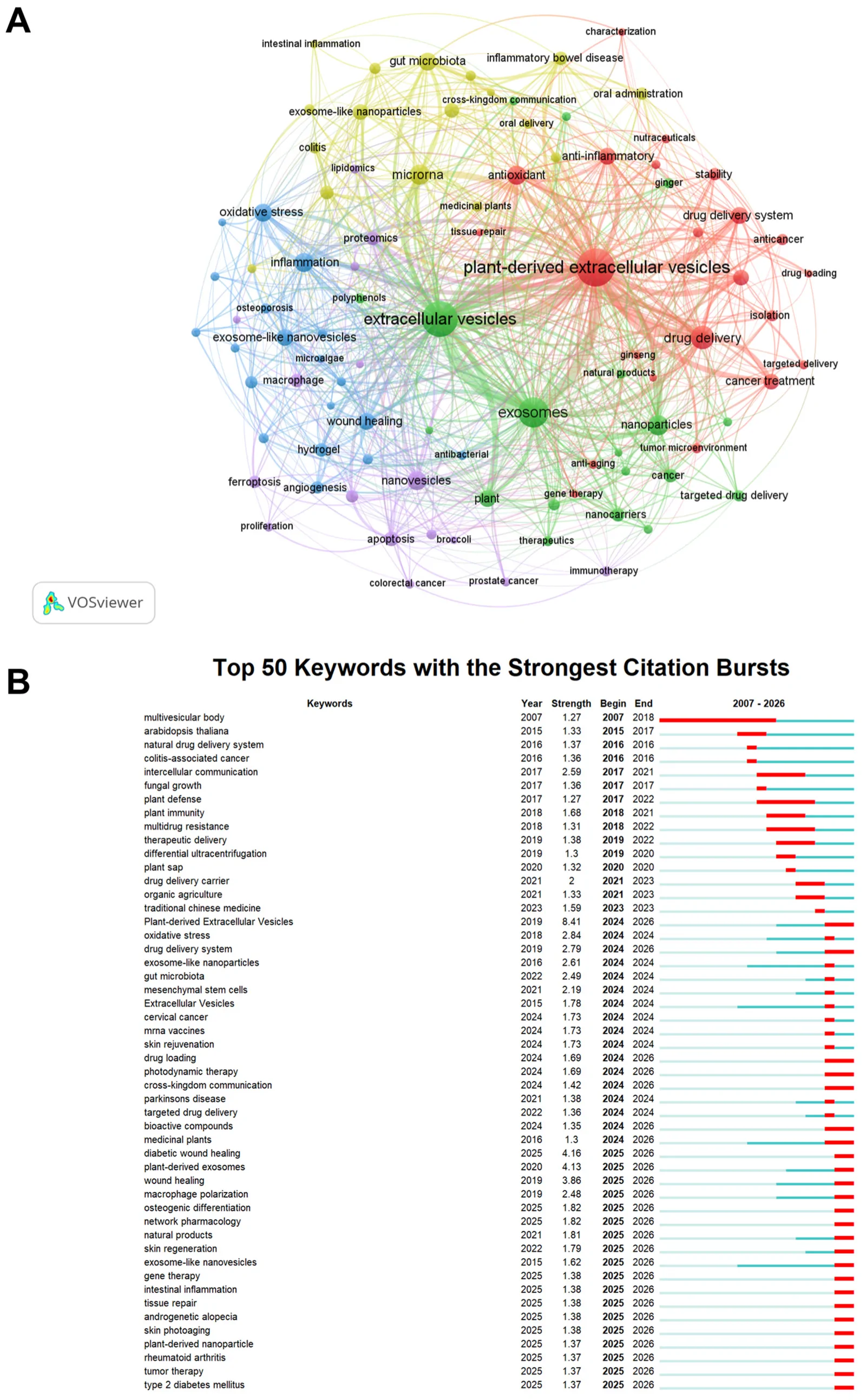
(**A**) Keyword co-occurrence network. In the network, the size of a node corresponds to its occurrence frequency, nodes in the same cluster share a color, and line thickness represents co-occurrence frequency, highlighting thematic connections. (**B**) Top 50 references with the strongest citation bursts.

We constructed a keyword co-occurrence network from 93 high-frequency terms (occurrence ≥ 6) to map the field’s intellectual structure and research priorities (Figure 4A). The term “plant-derived extracellular vesicles” serves as the network’s central node, with an occurrence of 336 and TLS of 268. It was followed by the foundational term “extracellular vesicles” (Occurrences = 271, TLS = 207) and the application-focused term “drug delivery” (Occurrences = 92, TLS = 87). The strongest co-occurrence link was observed between “drug delivery” and “extracellular vesicles” (link strength = 34), indicating that drug delivery is a predominant thematic emphasis.

Five thematic clusters were identified in this analysis. Null model testing confirmed their structural validity, with all q values < 0.05 (Supplementary Table S3). The network’s moderate density (0.191) and high clustering coefficient (0.564) further confirm the robustness of these cohesive thematic structures. The red cluster centers on “plant-derived extracellular vesicles” and includes “drug delivery”, “nanomedicine”, “targeted delivery”, “stability”, “drug loading”, “isolation”, “characterization”, “anticancer”, “blood-brain barrier”, and “tissue repair”. The co-occurrence structure of this cluster is predominantly associated with the isolation, characterization, and engineering concepts of PDEVs, with methodological terms co-occurring frequently with keywords related to oncology, tissue regeneration, and central nervous system disorders. The blue cluster centers on “inflammation” and includes “oxidative stress”, “macrophage polarization”, “mitochondria”, “antibacterial”, “periodontitis”, “osteoporosis”, “osteoarthritis”, “photoaging”, “wound healing”, “diabetic wound healing”, “skin regeneration”, “angiogenesis”, and “hydrogel”. The co-occurrence structure of this cluster reflects topics related to the anti-inflammatory, antioxidant, and immunomodulatory mechanistic concepts of PDEVs, with these terms co-occurring frequently with keywords related to chronic inflammatory diseases, metabolic disorders, tissue repair, and wound healing. The yellow cluster centers on “microrna” and contains “cross-kingdom regulation”, “gut microbiota”, “metabolomics”, “oral administration”, “inflammatory bowel disease”, “ulcerative colitis”, “colitis”, “neuroinflammation”, “ischemic stroke”, and “Parkinson’s disease”. The co-occurrence structure of this cluster reflects topics related to cross-kingdom communication involving the bioactive molecules of PDEVs, and these terms frequently co-occur with keywords related to the gut microbiota, gastrointestinal disorders, and neurological disorders. The purple cluster centers on “nanovesicles” and includes “proteomics”, “lipidomics”, “ferroptosis”, “breast cancer”, “prostate cancer”, “colorectal cancer”, “immunomodulation”, and “nlrp3”. The co-occurrence structure of this cluster reflects topics related to multi-omics characterization and molecular regulatory concepts of PDEVs, and these terms frequently co-occur with keywords related to oncology. The green cluster centers on “extracellular vesicles” and comprises “traditional Chinese medicine”, “ginger”, “herbal medicine”, “polyphenols”, and “natural products”. Detailed data are provided in Supplementary Table S4.

This study employed CiteSpace’s burst detection analysis to identify the top 50 keywords exhibiting the strongest citation bursts. As shown in Figure 4B, the keyword with the longest duration was “multivesicular body” (2007–2018), spanning over a decade. The highest burst intensities were recorded for “Plant-derived Extracellular Vesicles” (2024–2026, strength = 8.5), “diabetic wound healing” (2025–2026, strength = 4.16), “wound healing” (2025–2026, strength = 3.86), and “intercellular communication” (2017–2021, strength = 2.59). In the early stage (2007–2016), bursts were observed for “multivesicular body,” “arabidopsis thaliana,” “natural drug delivery system,” and “colitis-associated cancer,” signaling the field’s initial exploratory phase. From 2017 to 2023, prominent bursts included “intercellular communication,” “fungal growth”, “plant defense”, “differential ultracentrifugation”, “drug delivery carrier”, and “organic agriculture”, indicating a shift toward applied research with a particular focus on cross-kingdom signaling and regulatory interactions. From 2024 through 2026, bursts emerged across “drug delivery system,” “gut microbiota”, “drug loading”, “photodynamic therapy”, “oxidative stress”, “macrophage polarization”, “skin photoaging”, “Parkinson’s disease”, “diabetic wound healing”, “network pharmacology”, “gene therapy”, “androgenetic alopecia”, “mrna vaccines”, “osteogenic differentiation”, “rheumatoid arthritis”, “tumor therapy”, “intestinal inflammation”, and “tissue repair”, delineating the current focus on molecular mechanisms and translational applications.

In summary, the bibliometric analysis above delineates the intellectual structure of PDEV research. Building on these quantitative insights, the following sections synthesize key advances in isolation, purification, and multi-omics characterization. Specific attention is given to core regulatory mechanisms, including gut microbiota modulation and ferroptosis, followed by an evaluation of cutting-edge engineering strategies for drug loading and functionalization. Finally, these sections examine frontier therapeutic applications across oncology, inflammatory diseases, tissue regeneration, and neurological disorders, thereby providing a structured overview of the field’s current state and translational potential.

## 4. Review of Key Research Themes

### 4.1. Technical Cornerstones in PDEV Research

#### 4.1.1. Isolation and Purification Techniques

PDEVs have recently emerged as a significant research focus due to their unique biological roles and potential applications. Developing robust methods for their isolation and purification is essential for both fundamental research and translational applications. The International Society for Extracellular Vesicles (ISEV) has significantly contributed to standardizing extracellular vesicle research, with its updated MISEV2023 guidelines now serving as the field’s gold standard. These guidelines offer a systematic summary of EV isolation and purification techniques, encompassing principles for method selection, strategies to control contaminants, suggestions for technical refinement, and standards for standardized reporting [37]. However, the MISEV guidelines were developed almost entirely based on animal systems like mammals. Inherent biological differences mean applying these guidelines directly to plants brings significant challenges [38].

**Figure 5:**
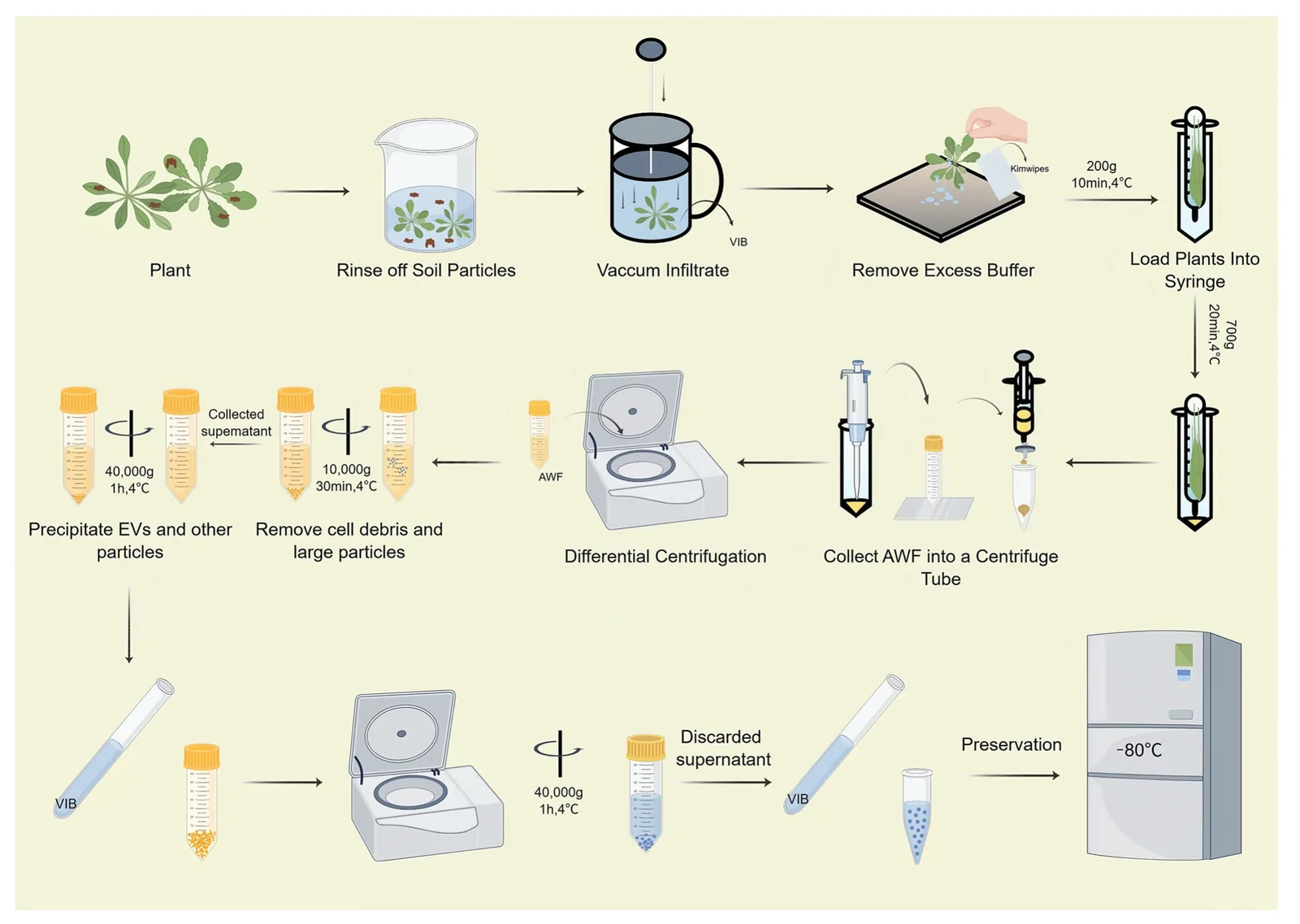
Isolation of EVs from Arabidopsis thaliana leaves by differential centrifugation. Rosettes of *Arabidopsis thaliana* were vacuum-infiltrated with vesicle isolation buffer (VIB) to obtain the apoplastic wash fluid (AWF). The AWF was subjected to sequential differential centrifugation: first at 10,000× *g* for 30 min to remove intact cells and large debris, followed by ultracentrifugation of the supernatant at 40,000× *g* for 1 h to pellet EVs and associated particles.

Establishing a standardized system for isolating, characterizing, and ensuring the quality control of PDEVs is crucial for future advancements. Mainstream methods for isolating and purifying PDEVs include differential centrifugation, size-exclusion chromatography, polymer precipitation, ultrafiltration, tangential flow filtration (TFF), and density gradient centrifugation [39,40,41,42,43]. Among these, differential centrifugation is widely considered the “gold standard” for EV isolation [44]. Emerging technologies include microfluidics and immunoaffinity capture [17]. Using differential centrifugation as an example, we detail the main procedures for isolating PDEVs from *Arabidopsis thaliana* [45] (Figure 5). As no single universal method satisfies all research needs, the optimal strategy is to choose an appropriate technique based on research goals and sample properties. Therefore, we summarize the principles, merits, and limitations of these methods to guide researchers in method selection (Table 3).

#### 4.1.2. Systematic Omics Technologies

The complex composition of PDEVs necessitates the application of multi-omics approaches like proteomics, transcriptomics, metabolomics, lipidomics to elucidate their biological functions. Proteomics employs liquid chromatography-tandem mass spectrometry to detect functional proteins in PDEVs, including defense-related enzymes and signaling molecules that aid in intercellular communication [73,74].

Transcriptomics uses high-throughput RNA sequencing (RNA-seq) to systematically analyze the RNA contents of PDEVs. This sequencing method facilitates the identification of diverse RNA types, including mRNA, lncRNA, circRNA, and miRNA, and predicts their potential target genes and signaling pathways in recipient cells. This approach clarifies the key roles that EVs play in regulating gene expression, metabolic processes, and cross-species signaling within recipient cells [75]. For example, Liu et al. employed RNA-seq and *Perilla*-specific small RNA-seq to show that *Perilla* leaf-derived PDEVs downregulate the IL-17 pathway in psoriatic lesions, with the endogenous pab-miR396a-5p identified as the key functional cargo [76].

Due to challenges such as low yields and difficulties in cargo characterization, metabolomics and lipidomics have been applied less frequently than proteomics and transcriptomics in PDEV research. Their analytical methods overlap considerably, utilizing common platforms like chromatography-mass spectrometry and nuclear magnetic resonance spectroscopy. Metabolomics focuses on the qualitative and quantitative analysis of secondary metabolites carried by PDEVs, highlighting the important roles these small molecules play in plants and cross-kingdom interactions. It can also track the dynamic changes in these metabolites in recipient cells, explaining how they are absorbed through signaling pathways and regulate metabolic networks within these cells [77,78]. Lipidomics enables the identification of the unique membrane lipid composition and structural characteristics of PDEVs, elucidating their roles in preserving vesicle stability, facilitating interspecies signal transmission, and enabling targeted delivery [79,80]. Nevertheless, single-omics analyses are insufficient to reveal the overall biological functions of PDEVs. Integrated multi-omics strategies enable researchers to construct a comprehensive functional landscape and systematic regulatory networks for PDEVs [81]. For example, Qi et al. employed multi-omics to elucidate how Chinese leek-derived extracellular vesicles (CL-EVs) alleviate muscle atrophy. Proteomics revealed enrichment of selenium-associated functional proteins in CL-EVs. Transcriptomic analysis of dexamethasone (DXM)-treated C2C12 cells combined with mining of public single-cell RNA-seq data from aging human muscle identified the AMPK/SIRT1/PGC-1α axis activation as the core mechanism for improving mitochondrial function and suppressing proteolysis. Complementary 16S rDNA sequencing and untargeted metabolomics further verified that CL-EVs exerted effects via the gut-muscle axis, restoring the Firmicutes/Bacteroidetes ratio and correcting metabolic disturbances in histidine metabolism and bile secretion in sarcopenic mice. This integrative multi-omics strategy provides solid evidence supporting the therapeutic potential of CL-EVs against sarcopenia [82].

### 4.2. Core Mechanistic Insights

#### 4.2.1. Gut Microbiota

**Figure 6:**
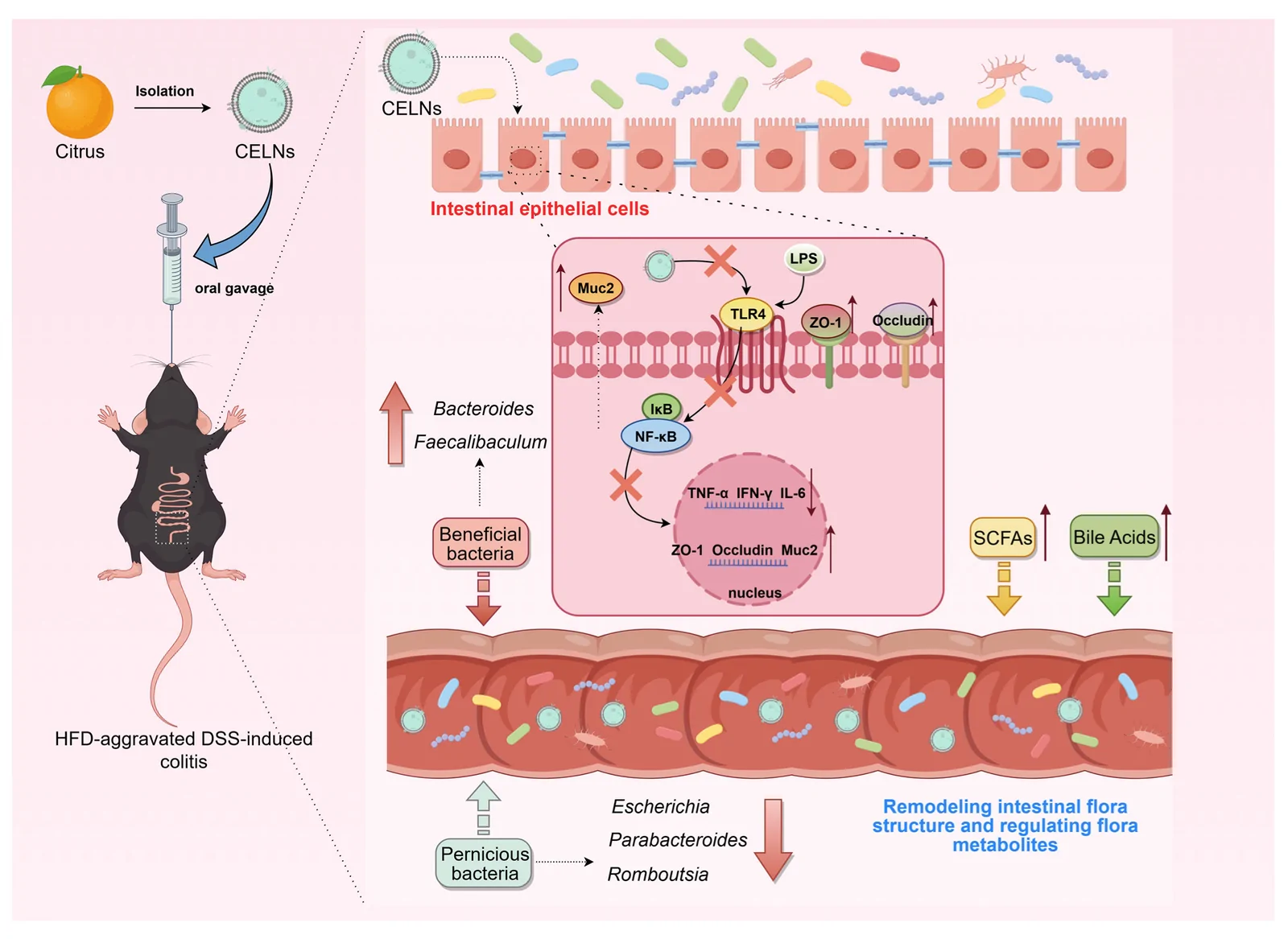
The mechanism of CELNs alleviating colitis aggravated by high-fat diet. After oral administration, CELNs enter the intestine and exert protective effects through the following ways: (1) remodeling the gut microbiota by increasing beneficial bacteria and reducing pathogenic bacteria; (2) modulating microbial metabolites by elevating SCFAs, restoring bile acid balance, and promoting indole derivative production; (3) repairing the intestinal barrier by upregulating tight junction proteins (ZO-1, Occludin) and mucin (Muc2); (4) suppressing the TLR4/NF-κB pathway, thereby reducing pro-inflammatory cytokines like TNF-α, IFN-γ, and IL-6.

Diet serves as the primary external influence on the gut microbiota, which is essential for maintaining host health [83,84,85,86]. A study by Mu et al. revealed that PDEVs withstand gastrointestinal degradation, reach the intestine intact, and modulate host cell functions through anti-inflammatory, antioxidant, and Wnt signaling pathways, thereby preserving intestinal homeostasis [29]. PDEVs exert a profound influence on the composition, metabolic functions, and microecological equilibrium of the gut microbiota (Figure 6). The primary mechanisms by which they regulate intestinal homeostasis are outlined below.

Firstly, PDEVs selectively enhance the growth of beneficial bacteria while suppressing pathogenic ones, thereby restructuring the microbial community composition. As demonstrated by Wang et al., orally administered garlic-derived exosome-like nanoparticles (GELNs) could be stably delivered to the intestine and internalized by gut bacteria. Additionally, the microRNA peu-MIR2916-p3, abundant in GELNs, promoted the proliferation of *Bacteroides thetaiotaomicron*, a bacterium with known anti-colitis benefits [87]. Tomato fruit-derived EVs not only promote the growth of probiotic *Lactobacillus*, but also significantly inhibit the growth of enteropathy-associated pathogens including *Fusobacterium nucleatum* and *Clostridioides difficile*, and reverse microbial dysbiosis induced by *Fusobacterium nucleatum* [88].

Second, PDEVs can modulate the metabolic activity of the gut microbiota and influence the production of microbial metabolites, thereby indirectly regulating the host’s metabolic homeostasis and immune status. For example, Zhan et al. demonstrated that oral administration of citrus exosome-like nanoparticles (CELNs) elevated the levels of short-chain fatty acids (SCFAs), essential metabolites produced by intestinal microbial fermentation [89]. Honeysuckle-Derived Nanovesicles (HNVs) elevate SCFA levels and regulate bile acid metabolism, thereby maintaining intestinal immune homeostasis [90]. Citrus-Derived Exosome-like Nanoparticles reshape the bile acid profile and promote anti-inflammatory indole derivatives, strengthening the intestinal barrier and suppressing inflammation [91]. Additionally, GELNs induce indole-3-propionic acid production via *Lactobacillus reuteri*, which correlates with reduced pro-inflammatory cytokines [92]. Lastly, PDEVs improve intestinal barrier function by influencing host cell activities. As demonstrated by Kim et al., orally administered ginseng-derived exosome-like nanoparticles (GENs) are absorbed by intestinal cells, leading to suppression of the NFκB pathway, which decreases proinflammatory cytokines and increases IL10 levels. GENs alter the gut microbiota by reducing the Firmicutes/Bacteroidetes ratio and enhancing *Lactobacillus* levels. These combined effects synergistically alleviate colonic crypt injury and inflammation, restoring intestinal barrier function and ameliorating colitis [93]. Similarly, *Andrographis paniculata*-derived exosome-like nanoparticles upregulate tight junction proteins such as Claudin-1, ZO-1, Occludin, Mucin2 and promote M2 macrophage polarization via PI3K-AKT and JAK-STAT pathways, restoring intestinal barrier integrity [94]. HNVs protect the mucosal barrier by increasing occludin, ZO-1, and Muc2 expression, elevating SCFAs, and rebalancing Treg/Th17 responses [90].

#### 4.2.2. Ferroptosis

Ferroptosis is an iron-dependent form of programmed cell death driven by lipid peroxidation. It arises from intricate crosstalk among cellular metabolism, oxidative stress, and iron homeostasis. Under physiological conditions, ferroptosis contributes to tumor suppression, immune regulation, and developmental processes. Pathologically, it is closely associated with multiple diseases including neurodegenerative disorders, organ injury, infections, and cancer, thus representing a critical potential therapeutic target [95,96,97].

Accumulating evidence indicates that PDEVs can modulate the ferroptosis pathway and demonstrate significant therapeutic potential. Their regulatory effects are bidirectional: they can either induce ferroptosis to suppress tumor growth, or inhibit it to protect normal tissues. For example, Zhang et al. demonstrated that *Pueraria lobata*-derived exosome-like nanovesicles (PLDENs) activated the NRF2/HO-1/GPX4 signaling axis upregulated hepatic GPX4 and GSH levels while downregulating ACSL4 expression, thereby reducing lipid peroxidation, before alleviating alcohol-induced liver damage in mice by inhibiting ferroptosis [98]. *Momordica charantia*-derived extracellular vesicle-like particles (MC-EVLPs) promote cancer cell apoptosis by modulating the Akt/Bcl-2/Bax pathway, reducing p-Akt levels and the Bcl-2/Bax ratio. Furthermore, MC-EVLPs markedly downregulate the key antioxidant protein GPX4 and upregulate the pro-ferroptotic proteins ACSL4 and transferrin receptor, leading to intracellular iron accumulation and elevated lipid peroxidation, thereby triggering ferroptosis and effectively inhibiting cervical cancer [99]. *Houttuynia cordata* Thunb-derived extracellular vesicle-like particles (HT-EVLPs) can naturally cross the blood–brain barrier and specifically target areas of cerebral infarction. By delivering endogenous miR-159a, HT-EVLPs directly bind and suppress the expression of the pro-ferroptotic gene ACSL4, thereby effectively inhibiting neuronal ferroptosis [100]. Carrot-derived exosomes (CAREX) loaded with the poorly soluble drug icariin (IC) form an IC-CAREX complex. IC-CAREX modulates the STAT3–GPX4 axis, and exerts potent antitumor effects against pancreatic cancer via a dual mechanism of inducing apoptosis and ferroptosis [101].

Beyond these classic examples, *Panax notoginseng*-derived nanovesicles (PNVs) disrupted redox homeostasis by inhibiting the p38–MAPK/NRF2 signaling pathway, leading to reactive oxygen species (ROS) accumulation and mitochondrial depolarization. This triggered the induction of ferroptosis by GPX4 downregulation and TFRC upregulation, autophagy, and PANoptosis [102]. Huangqi-derived exosome-like nanoparticles (HELNs) were shown to directly induce ferroptosis in prostate cancer cells by downregulating GPX4 protein expression, leading to Fe^2+^ accumulation and lipid peroxidation. Notably, HELNs were engineered via reversible electroporation to load siGPX4 (siGPX4@HELNs). This construct achieved more pronounced GPX4 knockdown, greater Fe^2+^ overload and lipid peroxidation, and superior tumor growth inhibition in vivo compared to HELNs alone, demonstrating the feasibility of combining PDEVs with genetic cargoes for precision ferroptosis therapy [103].

### 4.3. Engineering Platforms for Drug Delivery

#### Current Approaches for PDEVs Loading and Functionalization

PDEVs show great potential as drug delivery carriers owing to their advantages such as safety, biocompatibility, and low immunogenicity. For instance, Wang et al. first reported that Grapefruit-derived nanovectors have been shown to effectively load and transport chemotherapeutic drugs, nucleic acids, and proteins to target cells [35]. Despite the great potential of PDEVs, their practical applications remain limited by various challenges, including interspecies variability, insufficient targeting specificity, and low drug-loading efficiency [72]. Researchers are developing efficient drug-loading techniques and engineering strategies to enhance the therapeutic efficacy and targeting performance of PDEVs.

Currently, the commonly used drug loading strategies mainly include electroporation, co-incubation, and sonication. For example, Umezawa et al. employed electroporation to introduce exogenous microRNA (cel-miR-39) into PDEVs from various plants and exosomes from RAW 264.7 macrophages. They observed that electroporated PDEVs significantly improved the loading efficiency and protective effect of miRNA, allowing efficient miRNA delivery to the brain after intranasal administration in mice. Onion-derived nanoparticles loaded with miRNA via electroporation delivered dozens of times more miRNA to the caudal brain region within one hour after intranasal administration compared to the simple mixture control, and over 30 times more than mammalian cell-derived exosomes processed under the same electroporation conditions [104]. Electroporation loads drugs efficiently but irreversibly damages the PDEV lipid bilayers via high-voltage pulses. Sonication is milder yet requires precise power control—frequencies above 20 kHz rupture over 30% of vesicles, compromising yield. Extrusion, which forces vesicles through nanopores to homogenize size, causes far less membrane disruption. Zhang et al. [105] showed that extruding cholesterol-blended clematis PDEVs ten times yielded uniform vesicles (PDI < 0.2) with stable size and zeta potential over 28-day storage at 4 °C. The added cholesterol insertion further stiffened membranes, enhancing stability. However, excessive cycles (>20 passes) or temperatures above the lipid phase transition point risk stripping surface proteins, reducing steric hindrance, and triggering aggregation during storage. We have summarized the commonly used drug loading techniques and detailed their principle, type of loaded drug, advantages and limitations in Table 4.

**Figure 7:**
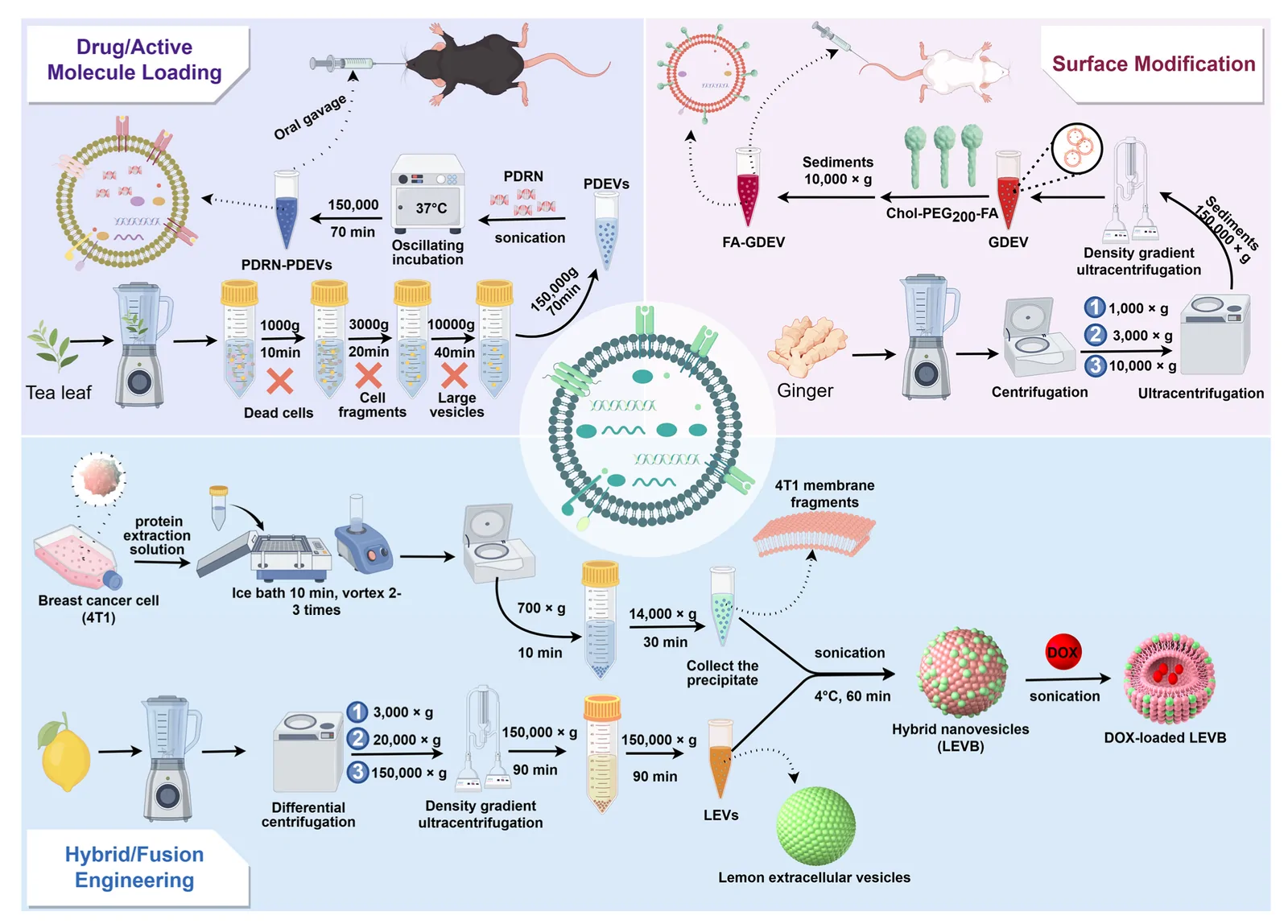
The preparation process of three PDEV-based engineering platforms for drug delivery. (1) Drug/Active Molecule Loading: Green tea-derived exosomes were isolated by differential centrifugation and ultracentrifugation, loaded with polydeoxyribonucleotide (PDRN) via sonication combined with oscillatory incubation. (2) Surface Modification: Ginger-derived exosomes were surface-conjugated with FA PEG_2000_ Chol and purified by density-gradient ultracentrifugation. (3) Hybrid/Fusion Engineering: LEVs were extracted by differential centrifugation and density-gradient ultracentrifugation, fused with 4T1 breast cancer cell membrane fragments via sonication to form hybrid nanovesicles (LEVB), and then loaded with DOX by sonication.

The common engineering strategies for PDEVs encompass physical modification, chemical modification, and membrane hybridization (Figure 7). Physical modification refers to the approach whereby functional molecules such as photosensitizers, thermosensitive agents, or specific ligands are introduced onto the surface of PDEVs via hydrophobic insertion or electrostatic adsorption [106,107,108]. For example, Han et al. anchored folic acid onto ginger-derived EVs via hydrophobic insertion of a cholesterol-PEG linker (FA-PEG_2000_-Chol), enabling targeted delivery to M1 macrophages in inflamed joints for rheumatoid arthritis therapy [109]. In addition to static surface decoration, stimuli-responsive drug loading strategies have recently gained attention. For example, Lu et al. reported that LED irradiation of PLDENs leverages their photosensitivity to produce ROS. The produced ROS temporarily and reversibly increased membrane permeability, thereby enabling efficient and controllable drug loading into the nanovesicles [110].

Chemical modification uses chemical reactions to attach functional molecules to PDEV membranes via stable chemical bonds, altering surface properties and boosting their targeting precision and stability [111]. Jamshidi et al. used EDC/NHS-mediated amidation to covalently attach anti-periostin peptide (APN) to the surface of lemon extracellular vesicles (LEVs), creating APN-modified LEVs (LEV-APN). The vesicles were loaded with doxorubicin (DOX) to create an effective targeted drug delivery system for breast cancer treatment [112]. Membrane fusion is a technique that uses physical methods to merge PDEVs with membrane structures from other specific cells. This process creates new vesicle carriers with hybrid membrane structures, giving PDEVs the biological activities and targeting abilities of the source cell membranes to enable precise drug delivery [113]. Yang et al., for instance, used sonication to fuse LEVs with 4T1 homologous tumor cell membranes, successfully creating hybrid nanovesicles. This strategy markedly improves the targeted delivery efficiency and therapeutic efficacy of the loaded drugs [114].

Our keyword analysis reveals that the frequency of the term “hydrogel” has increased markedly in recent years. This suggests that the construction of composite delivery systems by combining biomaterials such as hydrogels and liposomes with PDEVs, as an advanced biomimetic modification strategy, has become an important current research direction. Among these, hydrogels have emerged as ideal functional carriers owing to their high biocompatibility, tunable physicochemical properties, efficient drug loading and controlled release capacity, as well as favorable support for cellular activities [115,116,117]. For example, Yang et al. encapsulated titanium-doped cerium vanadatenanozymes with multi-enzyme activities into *Dendrobium officinale*-derived EVs. These were further incorporated into a dopamine-modified hyaluronic acid hydrogel to construct the DOEVs@CTT/DHM composite system. This system not only mimics natural enzyme activities to efficiently scavenge reactive oxygen species and promote angiogenesis, but also modulates mitophagy by activating the Nrf2/Parkin/Pink1 pathway, thereby restoring mitochondrial function. It provides a novel strategy for the application of hydrogel-PDEV composite systems in the precise regulation of organelle function for the treatment of refractory wounds.

Furthermore, unlike conventional engineering modification strategies, genetic modification techniques genetically edit parental cells to directly incorporate specific functional proteins onto exosome membranes or encapsulate them within exosome lumens during exosome biogenesis. This strategy has been well developed for mammalian cell-derived exosomes [118,119,120]. Currently, in the field of PDEVs, mature reports on similar genetic modification techniques remain scarce. This is largely limited by the presence of plant cell walls, the complexity of genetic manipulation, and the insufficient understanding of the biogenesis pathways of PDEVs. Nevertheless, the successful application of such strategies in animal exosomes has undoubtedly indicated a highly promising breakthrough direction. In contrast, the well-established genetic engineering toolbox for mammalian exosomes faces hurdles including high manufacturing costs and regulatory scrutiny over human/animal cell-derived products, making PDEV genetic modification a high-potential but technically challenging alternative.

**Table 4:** The main loading strategies for PDEVs.

Loading Strategy	Applicable Type	Advantage	Limitation	Ref.
Co-incubation	Hydrophobic small-molecule drugs	Extremely simple operation; minimal damage to exosomal membrane proteins and structure; no special equipment required	Generally low loading efficiency (usually < 5%); low diffusion efficiency for hydrophilic molecules; long incubation time (typically 12–24 h) and difficulty in precisely controlling drug loading.	[121,122,123]
Sonication	Hydrophobic/hydrophilic small molecules, nucleic acids, proteins, and other macromolecules	Extremely high loading efficiency (over 50%); broad applicability; significantly improves encapsulation of hydrophobic drugs	Requires expensive ultrasonic equipment; may cause exosome aggregation, morphological changes, or membrane protein denaturation; may lead to drug leakage under certain conditions.	[101,124,125]
Electroporation	Hydrophilic small molecules, nucleic acids, proteins, and other macromolecules	Relatively simple operation; highly efficient for loading macromolecules such as nucleic acids; short processing time.	Risk of impairing exosome integrity; prone to nucleic acid or exosome aggregation; relatively low loading efficiency especially for small-molecule drugs.	[120,126,127]
Extrusion	Hydrophobic small-molecule drugs	Significantly improves drug encapsulation; enables preparation of exosome–liposome hybrid vesicles; high loading efficiency.	High equipment requirements (high-pressure extruder); strong shear force may cause morphological changes or membrane protein shedding; difficulty in precisely controlling the drug-to-exosome ratio.	[105,124]
Freeze–Thaw Cycles	Hydrophilic molecules and antioxidants	Simple operation; suitable for large-scale preparation; good protection of bioactivity.	Low loading efficiency (usually < 30%); easy to induce exosome aggregation or irreversible structural damage; considerable impact on membrane protein stability	[128,129,130]

### 4.4. Frontier Therapeutic Applications

#### 4.4.1. Cancer Therapy

**Figure 8:**
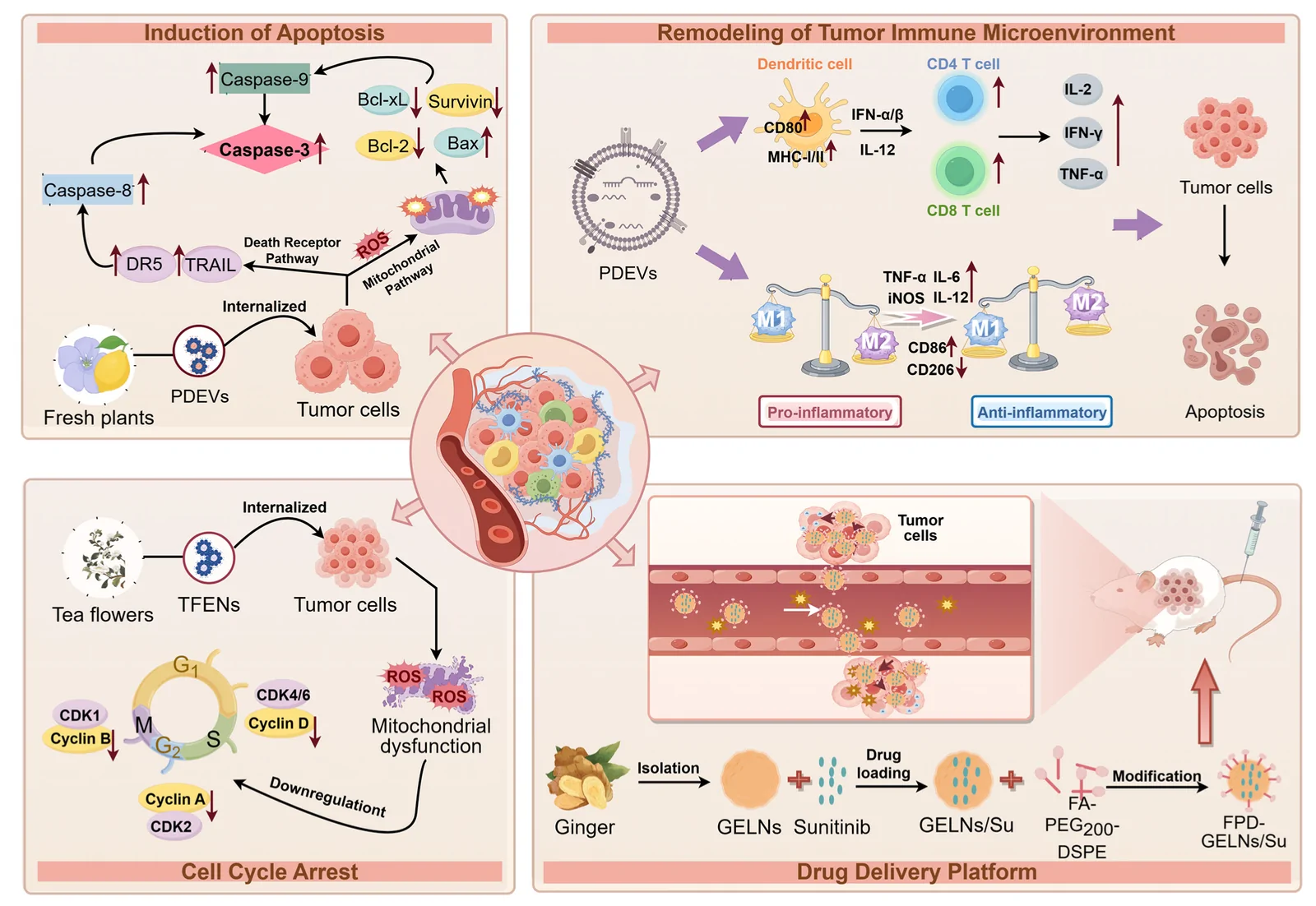
Potential mechanisms of PDEVs in tumor therapy.

Addressing the urgent need for precise targeting and low toxicity in cancer therapy has long been a formidable challenge. PDEVs offer new hope for cancer treatment due to their safety and high efficiency in drug delivery, and intrinsic therapeutic activity. Current research indicates that the anticancer effects of PDEVs are achieved through four major mechanisms: regulating apoptosis, arresting the cell cycle, reprogramming the tumor microenvironment, and mediating targeted delivery (Figure 8).

PDEVs predominantly induce tumor cell apoptosis by impairing mitochondrial function and activating extrinsic death receptor pathways. Regarding the intrinsic apoptotic pathway, Yang et al. found that PDEVs derived from *Platycodon grandiflorum* were effectively taken up by triple-negative breast cancer cells, causing a marked increase in intracellular ROS levels. This subsequently initiated apoptosis via the cascade activation of key executioner caspases, including Caspase-9 and Caspase-3 [131]. Complementarily, the extrinsic apoptotic pathway involves the upregulation of death receptor signaling. For instance, Lemon juice-derived nanovesicles significantly upregulate the expression of TRAIL and its receptor DR5 on the surface of tumor cells, thereby promoting the secretion of TRAIL protein to exert their anti-tumor effects [36].

PDEVs impede tumor cell proliferation by arresting the cell cycle at the G0/G1, S, and G2/M phases [132,133]. For instance, Onion-derived exosome-like nanoparticles were efficiently taken up by MNNG-induced malignantly transformed gastric mucosal epithelial cells, and exerted antitumor effects by downregulating the expression of PCNA, a key protein involved in the S phase of the cell cycle [134]. Furthermore, PDEVs also exert antitumor effects by modulating key oncogenic signaling pathways such as PI3K/Akt/mTOR, MAPK/ERK, STAT3/PD-L1, Wnt/β-catenin, and lipid metabolism [135,136,137]. For example, *fagopyrum dibotrys*-derived nanovesicles can activate the p53/xCT/GPX4 signaling axis, leading to the accumulation of lipid peroxidation products, induction of ferroptosis, and inhibition of tumor growth [96].

PDEVs primarily exert their anticancer effects by remodeling the tumor microenvironment through the regulation of macrophage polarization and enhancement of T cell activity. Moreover, they can also act on cancer cells, endothelial cells, epithelial cells, fibroblasts, and Treg cells [138,139,140]. Han et al. demonstrated that *Eutrema japonicum*–derived exosome-like nanoparticles effectively promoted dendritic cell maturation, drove the differentiation of naive CD4+ T cells into IFN-γ-producing Th1 cells, and significantly activated cytotoxic CD8+ T cells, ultimately leading to pronounced tumor growth inhibition [41]. Liu et al. demonstrated that *Pinellia* exosomal vesicles are absorbed by macrophages, transforming pro-tumor M2 macrophages into anti-tumor M1 macrophages by inhibiting serine synthesis and activating JAK-STAT signaling. Additionally, these vesicles directly induce S-phase cell cycle arrest in lung cancer cells, thereby effectively suppressing tumor growth [141]. In contrast to synthetic nanomaterials, which often provoke nonspecific inflammatory responses due to surface charge or compositional issues, PDEVs leverage their inherent immunomodulatory properties. This not only circumvents such adverse effects but also synergistically enhances the host’s antitumor immunosurveillance, underscoring their superior biosafety and biocompatibility over synthetic carriers.

PDEVs can deliver natural bioactive components like specific miRNAs, lipids, and phytochemicals to mammalian cells, functioning as a delivery system to achieve precise targeting and exert antitumor effects. In addition, PDEVs also effectively carry chemotherapeutic drugs and nucleic acids, thereby enhancing drug accumulation at tumor sites, reducing systemic toxicity, and overcoming multidrug resistance [142,143,144]. For example, LEVs loaded with the chemotherapeutic prodrug capecitabine increased the concentration of 5-fluorouracil, the active metabolite of capecitabine, in tumor tissues to approximately 13-fold that of the free capecitabine group [145]. Xu et al. found that loading sunitinib into ginger-derived nanoparticles modified with folic acid-polyethylene glycol improved uptake by renal cancer cells. This method downregulated both the mRNA and protein expression levels of ABCB1/P-glycoprotein, a key transporter responsible for drug efflux and multidrug resistance, thereby effectively reducing sunitinib efflux from cancer cells and increasing its intracellular accumulation, reversing the resistance of renal cancer cells to sunitinib and enhancing its anticancer efficacy [146]. Duan et al. showed that doxorubicin delivered by *Rhodiola rosea*-derived exosome-like nanovesicles not only exhibited superior tumor-targeting ability and growth inhibition compared to free doxorubicin, but more importantly, significantly mitigated the hallmark cardiotoxicity of doxorubicin. These findings indicate that PDEVs, as drug delivery carriers, can effectively reduce organ toxicity while enhancing chemotherapeutic efficacy [147].

#### 4.4.2. Inflammatory Diseases Therapy

PDEVs hold great promise for preventing and treating inflammatory diseases, offering new hope for precise, multi-targeted therapies. PDEVs exert these effects by serving as delivery vehicles for their intrinsic bioactive cargo and by modulating the immune system and host microbial homeostasis [148,149,150]. For instance, Shi et al. demonstrated that *Centella Asiatica*-derived exosomes (CAEs) selectively target and accumulate in the inflamed colon after oral administration, achieving precise delivery. The plant miRNAs in CAEs are taken up by intestinal immune cells, mitigating local inflammation by modulating macrophage polarization and inhibiting key inflammatory pathways [151].

Recent studies have employed advanced techniques such as surface modification, membrane fusion, and innovative formulations to engineer PDEVs, elucidating novel mechanisms and targets including cellular metal ion homeostasis, organelle function regulation, and targeted modulation of specific gut bacteria. For instance, Han et al. found that in rheumatoid arthritis, the gut bacterium *Ruminococcus gnavus* aggravates arthritis by secreting phenylethylamine (PEA), which activates the PEA-BTK axis and induces excessive neutrophil extracellular trap formation. They further discovered that PLDENs are preferentially taken up by *R. gnavus*. The lipid components of PLDENs facilitate the intestinal enrichment of the plant miRNA gma-miR4412, which directly targets and inhibits bacterial phenylalanine decarboxylase gene expression, thereby reducing PEA production and ultimately alleviating arthritis by blocking the PEA-BTK-NETs axis [152].

In a study by Cao et al., tea-derived EVs loaded with PDRN were developed as a nanoplatform (PDRN-EVs) for targeted oral delivery to colonic inflammatory sites. PDRN-EVs were specifically taken up by macrophages and shifted them from a pro-inflammatory M1 to an anti-inflammatory M2 state by activating the cAMP/HIF-1α pathway, thereby reducing TNF-α levels and increasing IL-10. Furthermore, orally administered PDRN-EV restored gut microbiota dysbiosis in DSS-induced colitis mice, increasing the abundance of beneficial bacteria such as *Muribaculaceae* [153]. Wang et al. constructed a multi-layered nanoplatform (CS-Dex/HD@REVs) using rhubarb-derived extracellular vesicles (REVs) as the core, sequentially coated with a redox-responsive drug-loaded polymer middle layer and a chondroitin sulfate-dextran conjugate outer shell. This design achieved colon-specific accumulation via dextranase responsiveness, active targeting of CD44 receptors on inflammatory macrophages through chondroitin sulfate, and triggered release of DXM in the high-ROS microenvironment, synergizing with the intrinsic therapeutic activity of REVs [154].

Conversely, Zhang et al., on the other hand, used UV irradiation as an external stressor to pretreat the raw plant material, increasing the yield of Morinda officinalis-derived nanovesicles (MOE) by 2- to 3-fold and significantly enhancing their antioxidant activities. They then camouflaged MOE with erythrocyte membranes to construct MOE@EM, which reduced immunogenicity, improved in vivo circulation and joint targeting, and effectively accumulated in the arthritic joints of model mice. MOE@EM promoted macrophage repolarization from the pro-inflammatory M1 to the anti-inflammatory M2 phenotype by inhibiting the Wnt/β-catenin pathway. Furthermore, it significantly suppressed dendritic cell activation and reduced the numbers of pro-inflammatory Th1 and Th17 cells within the joints, thereby remodeling the immune microenvironment at multiple levels and ultimately alleviating bone erosion and joint swelling [155]. Table 5 summarizes the therapeutic effects and mechanisms of PDEVs from different sources in a variety of inflammatory diseases.

#### 4.4.3. Tissue Regeneration, Repair, and Aesthetic Applications

**Figure 9:**
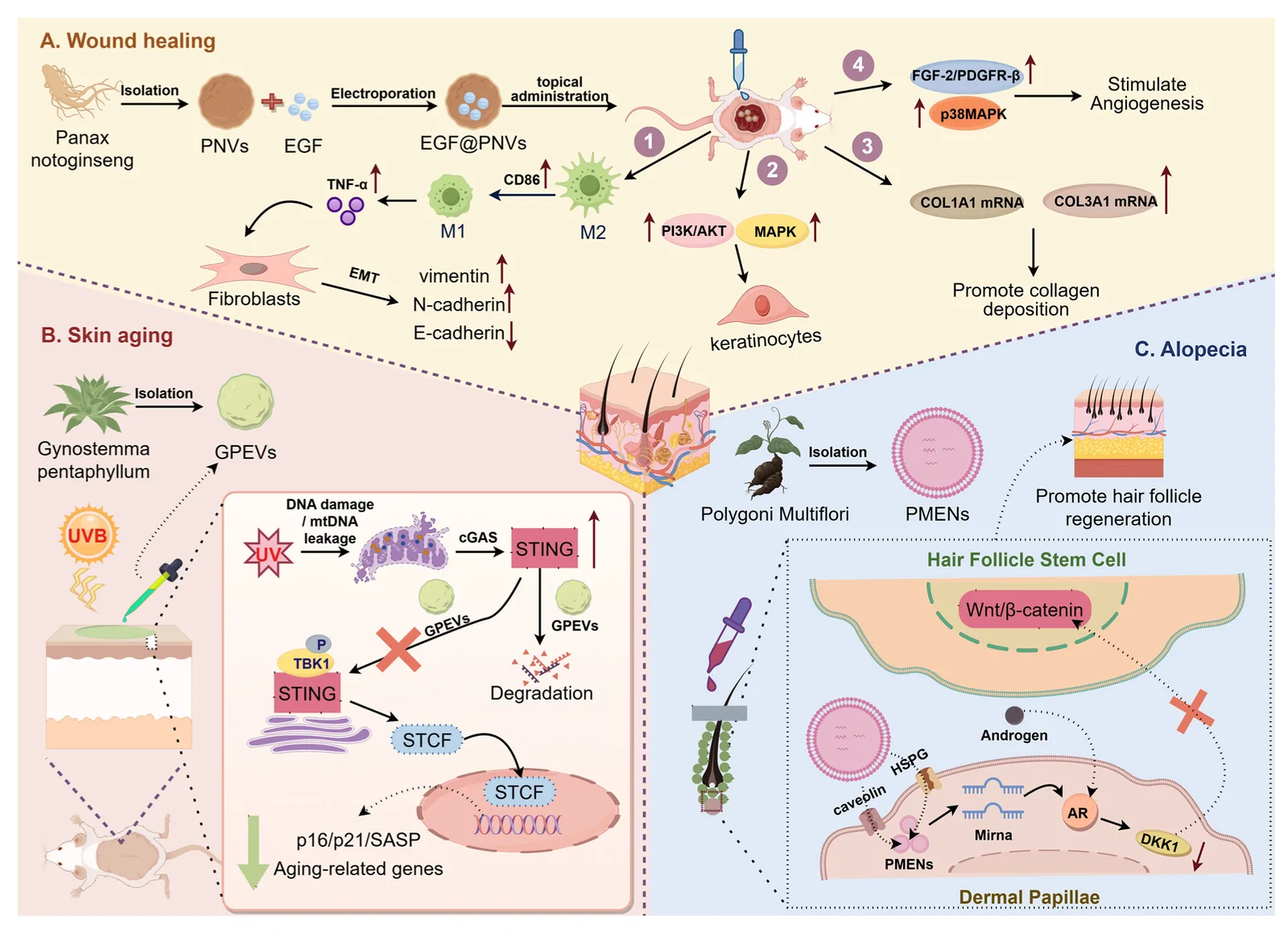
Mechanisms of PDEVs in maintaining skin health. (**A**) EGF-loaded PNVs accelerate wound healing by promoting collagen synthesis and angiogenesis. (**B**) Gynostemma pentaphyllum-derived extracellular vesicles (GPEVs) alleviate UVB-induced photoaging via STING degradation. (**C**) Polygonum multiflorum-derived exosome-like nanovesicles (PMENs) promote hair follicle regeneration by inhibiting the androgen receptor (AR) and activating the Wnt/β-catenin pathway.

PDEVs have attracted considerable attention in skin health, demonstrating promise in applications from wound healing to aesthetic enhancement (Figure 9). Wound healing is a biological process through which damaged skin restores its structural integrity and barrier function, primarily involving four overlapping phases: hemostasis, inflammation, proliferation, and remodeling [180]. Due to their low immunogenicity, biocompatibility, and abundance of bioactive molecules, PDEVs have shown remarkable promise in repairing various tissue injuries such as skin, bone, nerve, and cardiovascular system, thereby addressing key limitations of conventional therapies such as limited efficacy, high cost, and immune rejection [181,182,183].

PDEVs exert therapeutic effects via multiple mechanisms, including immune modulation, regulation of cellular behavior, promotion of angiogenesis, antioxidant activity, antibacterial activity, and modulation of the microbiota [184,185,186]. For instance, Tan et al. showed that ginseng-derived exosomes (GExos) effectively promote angiogenesis by reprogramming endothelial cell metabolism. GExos enhance glycolysis by upregulating key enzymes (PFKM, PGK1, ENO1), facilitating glucose conversion, and supporting cell proliferation and migration. They also lower acetyl-CoA levels, suppress oxidative phosphorylation, and reduce mitochondrial ROS, mitigating oxidative stress in high-glucose environments. This restores endothelial cell function, significantly improving angiogenesis and wound healing in diabetic skin ulcers [187]. Huang et al. demonstrated that *Salvia miltiorrhiza*-derived extracellular vesicles (SMEVs) significantly downregulated the mRNA expression of pro-inflammatory cytokines such as IL-1β, IL-6, and TNF-α in LPS-stimulated endothelial cells. Meanwhile, SMEVs were efficiently internalized by HUVECs, promoting endothelial cell migration and wound healing. SMEVs also restored the vascular endothelial barrier by enhancing VE-cadherin and CD31 expression in lung tissue. Notably, this study showed that SMEVs modulated pulmonary microbiota diversity and richness, reduced pathogens like *Listeria* and *Streptococcus*, and reshaped the lung microbial community after LPS stimulation [48].

PDEVs possess inherent bioactivity and can be engineered to carry therapeutic molecules, enhancing their stability, bioavailability, and targeting, thus aiding in combination therapies for complex diseases [188,189,190]. Additionally, AI is being used to efficiently identify wound-healing components from PDEVs [191]. For instance, Gao et al. constructed a core–shell nanoplatform (Z-Lyc@ELP) based on GELNs. The shell, composed of unsaturated phospholipid-doped GELNs and polymyxin B (PMB), endows the platform with ROS responsiveness, while the core consists of a ZIF-8 framework loaded with lycopene (Lyc) and exhibits pH-responsive degradation. In the early infection stage, high ROS levels at the lesion site cause the oxidative breakdown of unsaturated phospholipids in the Z-Lyc@ELP shell, leading to its disintegration. This releases PMB to kill bacteria and natural immunomodulatory molecules from GELNs to reduce inflammation. Leaked Lyc enhances antibacterial effects by disrupting bacterial membranes and interfering with oxidative stress defense. As the infection progresses and the lesion becomes more acidic, the ZIF-8 core degrades, releasing Lyc and Zn^2+^ ions. These components scavenge residual ROS, promote macrophage M2 polarization, and upregulate pro-angiogenic factors like VEGFR, providing antioxidant, anti-inflammatory, and tissue-repair benefits in the later stage [184].

Although wound healing typically results in scarring, there is a strong demand for skin that heals without marks and maintains its youthful appearance. This has led scientists to investigate PDEVs for their potential in anti-aging and skin beautification. Skin aging is primarily driven by oxidative stress, inflammation, extracellular matrix degradation, and cellular senescence. PDEVs exert anti-aging and skin-beautifying effects through multiple pathways, including regulating signaling pathways, delivering bioactive molecules, restoring the extracellular matrix, and improving skin barrier function [192,193,194]. For example, Li et al. showed that lavender-derived exosome-like nanoparticles (LELNs) deliver plant miRNAs, specifically cpa-miR166e and zma-miR166h-3p, which regulate inflammation and collagen metabolism. These miRNAs are involved in pathways such as DNA repair and autophagy. LELNs reduced UVB-induced inflammation and matrix metalloproteinase levels while enhancing type I collagen synthesis in cell cultures and mouse skin, and effectively mitigated UVB-induced photoaging [195]. Lin et al. demonstrated that miRNAs from tea leaf-derived nanovesicles can exert cross-kingdom regulation by targeting and inhibiting the MYB4 transcription factor in mammalian cells. This activation of the PI3K/AKT pathway leads to a significant reduction in MITF and tyrosinase, effectively inhibiting melanin production, highlighting the exceptional whitening effects of PDEVs in both cell and animal models [196].

Furthermore, PDEVs can be modified through surface engineering to enhance their targeting specificity toward particular skin cells, or combined with advanced formulations to create smart, sustained-release, moisture-retaining systems, enhancing topical application convenience and extending therapeutic effects. For example, Wang et al. developed a hybrid nanoplatform by fusing ginseng root-derived exosomes with proanthocyanidin-loaded liposomes using microfluidic membrane fusion technology. Importantly, this study combined the exosome-based nanoplatform with microneedle roller technology, an advanced physical delivery technique. By actively creating microchannels, this approach effectively overcame the stratum corneum barrier and significantly enhanced the penetration and retention of the nanoformulation in the deep skin layers. In both a chronic UV-induced photoaging mouse model and a human study, the system safely and effectively improved skin wrinkles, elasticity, and hydration by prolonging local skin retention and enabling sustained drug release [197].

These findings demonstrate the immense potential of PDEVs, when engineered and integrated with advanced formulations, for achieving convenient and durable topical skin therapy. Notably, PDEVs have also shown potential in promoting hair regeneration and improving the function of skin appendages. For instance, ginger-derived exosome-like nanoparticles were engineered to load finasteride and incorporated into a thermosensitive hydrogel for topical treatment of androgenetic alopecia. This integrated system enhanced transdermal drug retention, lowered local dihydrotestosterone levels, promoted hair follicle transition from telogen to anagen via upregulating VEGF and Ki67, and stimulated robust hair regeneration [198].

#### 4.4.4. Neurological Disorders Therapy

**Figure 10:**
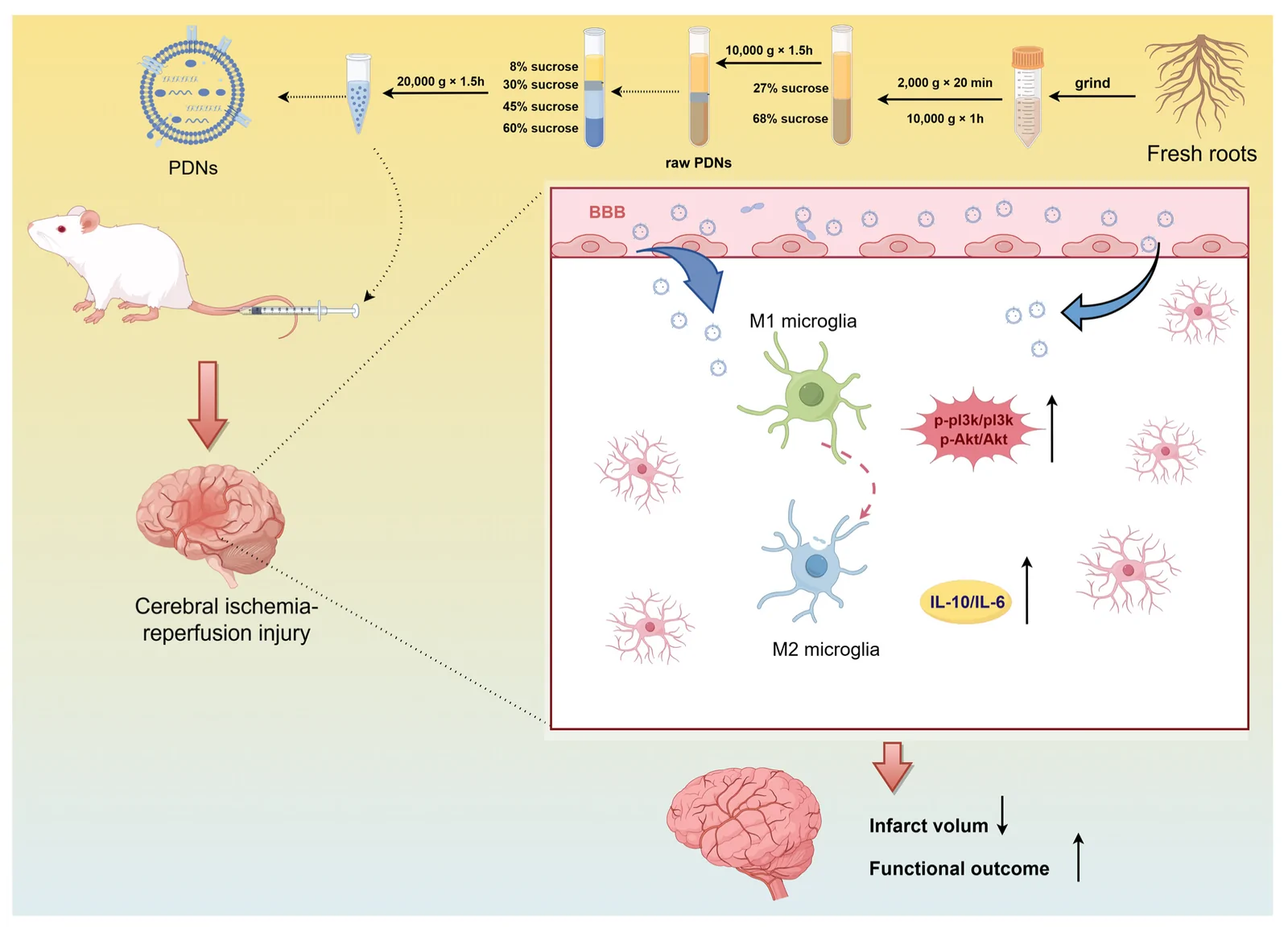
Schematic illustration of PDN-mediated neuroprotection. PDNs are isolated from *Panax notoginseng* via sucrose gradient ultracentrifugation. Upon intravenous injection, PDNs extravasate across the compromised BBB and are preferentially internalized by microglia, polarizing them toward the M2 phenotype and ultimately reducing cerebral infarct volume.

The blood–brain barrier (BBB) is a major obstacle in the treatment of neurological disorders. PDEVs are attracting significant attention due to their innate ability to pass through biological barriers and serve as multifunctional drug delivery systems for neurological diseases (Figure 10). For instance, Kim et al. demonstrated that GENs can serve as natural nanocarriers to deliver plant-derived miRNAs for silencing the oncogene c-MYC. GENs efficiently penetrate the BBB, achieve efficient accumulation at the lesion site, modulate the tumor microenvironment, and induce macrophage phenotype polarization, thereby significantly inhibiting glioma growth [199].

Through engineering strategies such as surface modification, drug loading, and membrane fusion, recent studies have enhanced the translational potential of PDEVs for treating complex neurological disorders [200]. For example, in a study by Xu et al., *Pueraria lobata*-derived exosomes modified with DSPE-PEG-RVG efficiently traversed the BBB in a Parkinson’s disease model and accumulated in the brain. By promoting PINK1/Parkin-mediated mitophagy and improving mitochondrial function, they significantly enhanced dopaminergic neuron survival, thereby reducing both motor and non-motor symptoms in mice [201]. In a study, Apple-derived exosome-like nanoparticles (Apple ELNs) were coated with polydopamine (PDA) and functionalized with gadolinium ions (Gd^3+^) to form PDA@ELNs/Gd. In a mouse model of ischemic stroke, PDA@ELNs/Gd achieved superior penetration of the blood–brain barrier and targeted concentration in the ischemic brain region compared to unmodified ELNs. Furthermore, the loaded Gd^3+^ endowed the modified Apple ELNs with the ability to serve as an MRI contrast agent, enabling non-invasive visualization and diagnosis of ischemic lesions [202]. Thus, the main advantage of PDEVs in treating neurological disorders is their capability to cross the BBB naturally or through engineering, allowing them to target multiple issues in both acute injuries and chronic diseases by utilizing their inherent bioactivity and cross-kingdom regulation.

Ischemic stroke results from the disruption of blood supply to local brain tissue due to vascular occlusion. Owing to their unique biological properties, PDEVs offer innovative solutions to overcome the time window limitations of conventional therapies represented by intravenous thrombolysis and mechanical thrombectomy, the risk of reperfusion injury, and the challenge of blood–brain barrier penetration for neuroprotective agents. For example, *Momordica charantia*-derived exosome-like nanovesicles have been shown to effectively ameliorate hemorrhagic transformation exacerbated by delayed tissue plasminogen activator (t-PA) thrombolysis, offering new possibilities for safe thrombolytic therapy beyond the conventional 4.5 h time window [203]. Exosome-like nanoparticles derived from *Panax notoginseng* (PDNs) penetrated the brain and shifted microglia from the pro-inflammatory M1 state to the anti-inflammatory M2 state, stimulating the PI3K/Akt pathway, thereby markedly alleviating cerebral ischemia/reperfusion injury [204]. Celery seed-derived reconstituted lipid nanoparticles (CS-rLNPs) demonstrate anti-inflammatory and anti-apoptotic effects by acting on the TLR4/MyD88/NF-κB p65 pathway. Moreover, CS-rLNPs have been demonstrated to possess innate neuron-targeting ability, primarily binding to neurons in the ischemic penumbra [205].

Furthermore, *Panax notoginseng*-derived extracellular vesicle-like nanoparticles delivered plant microRNAs to recipient neurons, mediating cross-kingdom regulation of host cell stress granule dynamics and mTOR signaling, thereby restoring neuronal function and mitochondrial homeostasis [206]. Notably, orally administered PDEVs can also reshape the intestinal microenvironment by modulating gut microbiota, repairing the intestinal barrier, and regulating intestinal immunity, thereby generating systemic anti-inflammatory and reparative signals that ultimately act synergistically on the brain to protect BBB integrity and promote neural repair. For example, orally administered bitter melon-derived exosome-like nanoparticles can effectively traverse the intestinal barrier, mediate the entry of beneficial gut microbial metabolites into the systemic circulation, and cross the blood–brain barrier to target the ischemic brain region. Within the brain, they downregulate MMP-9 expression, upregulate tight junction proteins (ZO-1, claudin-5), and inhibit neuronal apoptosis via activation of the AKT/GSK3β signaling pathway, thereby cooperatively protecting blood–brain barrier integrity, attenuating neuroinflammation, and facilitating neural repair [207].

The potential benefits of PDEVs in the treatment of neurodegenerative diseases like Alzheimer’s and Parkinson’s have attracted more attention in recent years. For instance, EVs isolated from the medicinal plant *Gardenia jasminoides* were shown to alleviate rotenone-induced mitochondrial dysfunction and apoptosis in dopaminergic neurons by inhibiting the p38 MAPK/p53 signaling pathway, thereby effectively reducing α-synuclein levels, protecting dopaminergic neurons, and improving motor function in both PC12 cells and *Caenorhabditis elegans* models [208]. In a study by Cao et al., EVs derived from the roots of the traditional Chinese medicinal herb Drynariae Rhizoma were analyzed by proteomics, identifying 77 proteins—among which enzymes accounted for up to 47%. Further KEGG pathway enrichment revealed that these enzyme proteins were not only enriched in the human oxidative phosphorylation pathway but also significantly associated with pathways related to Alzheimer’s disease, Huntington’s disease, and Parkinson’s disease [209].

Additionally, REVs were found to significantly reduce abnormally elevated ROS levels in a fibroblast model derived from Alzheimer’s disease patients. They also modulated cellular energy metabolism by specifically decreasing the aberrantly high glycolytic flux in AD cells, helping to restore metabolic homeostasis [210]. These findings offer a justification for the development of Rhubarb-derived EVs as natural nutritional supplements or therapeutic carriers with neuroprotective potential.

## 5. Discussion

The marked acceleration in publications on PDEVs since 2017 marks a clear transition from a nascent exploratory phase to a dynamic research hotspot. This trend can be attributed to breakthroughs and commercialization in exosome isolation technologies, which have lowered technical barriers and enabled most laboratories to conduct relevant research. Meanwhile, PDEVs have been demonstrated to exhibit multiple potential therapeutic effects. Their wide availability, low cost, and high safety profile confer substantial scientific significance and promising commercial potential [211,212,213,214].

**Figure 11:**
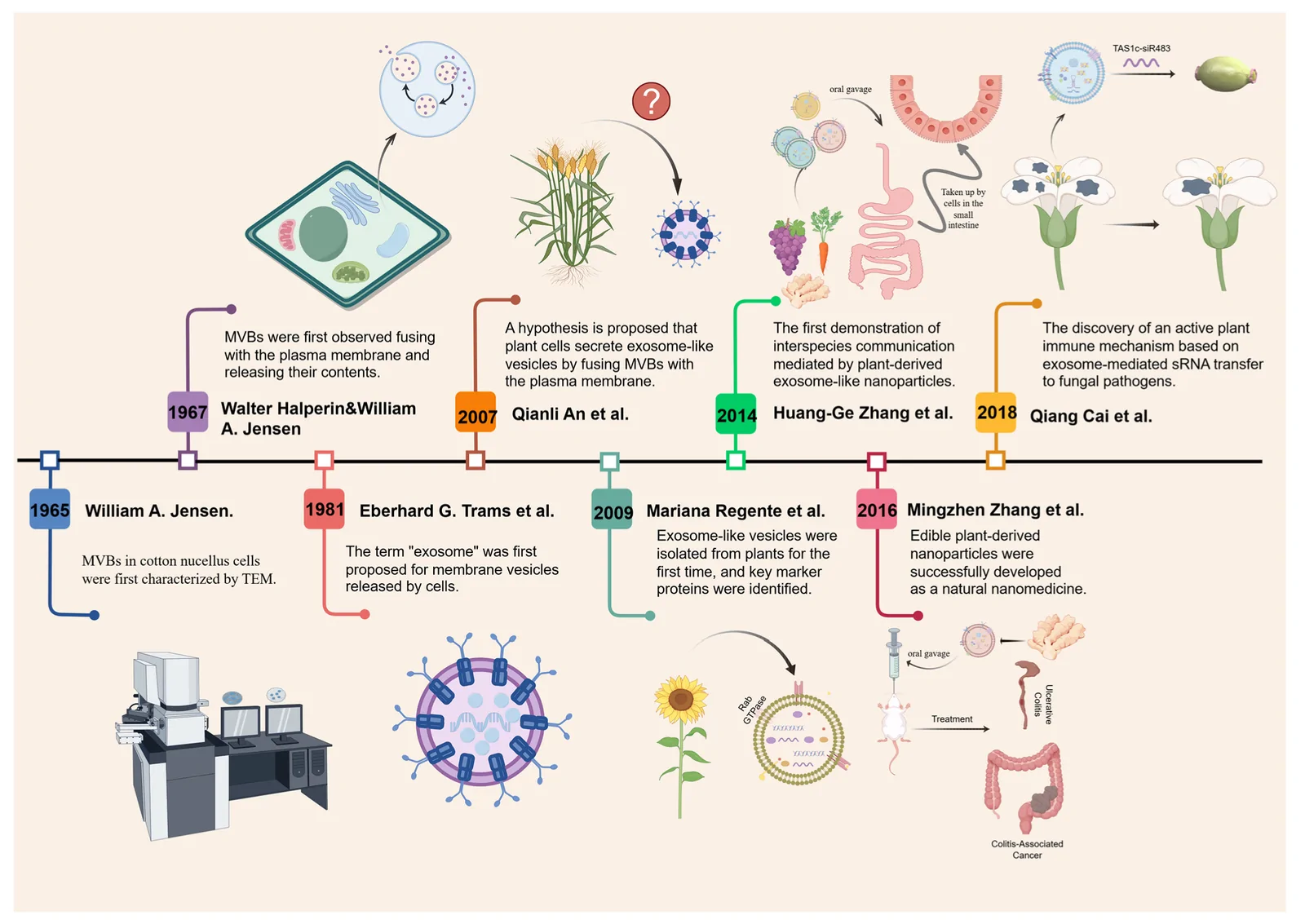
Evolution of the PDEV field: Chronological timeline of key contributors and findings [4,16,27,28,30,59,218,219].

Consistent with these trends, our co-citation analysis identified a stable knowledge backbone comprising seminal works primarily from 2013 to 2018. These pioneering studies lay the essential groundwork for understanding interspecies communication mediated by PDEVs. They elucidated key mechanistic pathways, established reliable isolation and characterization methods, and demonstrated the therapeutic potential of PDEVs. Furthermore, a highly cited study by Cai et al. (2018) [30] transformed the view of PDEVs from simple plant extracts with bioactive components to carriers of functional molecules with cross-kingdom effects, elevating them from a niche topic to broader academic interest and establishing a foundation for their use in drug delivery and cross-kingdom regulation research. Subsequently, we compiled a chronological timeline that highlights landmark contributions along with the associated researchers (Figure 11).

Based on the keyword analysis results, we have identified important research hotspots and frontiers in this domain, conducted an extensive review to highlight the latest advancements and breakthroughs. PDEVs have demonstrated promising application prospects in the biomedical field, offering hope for various intractable diseases. However, this field still faces several challenges and future breakthrough directions, particularly in large-scale production, standardized preparation, and in-depth understanding of biological mechanisms, which will be discussed in detail below.

1. The lack of unified international nomenclature standards has led to widespread interchangeable use of terms including “plant exosomes”, “plant-derived exosome-like nanoparticles” and “plant nanovesicles” across the field. This complicates literature searches and data interpretation, weakens inter-study comparability, and obscures mechanistic attribution. Critically, current studies often conflate two vesicle populations with distinct biogenesis pathways: plant extracellular vesicles (PEVs) actively secreted by living cells, and plant-derived nanovesicles (PDNVs) obtained via mechanical tissue disruption, which differ markedly in biogenesis, composition and function [215]. The biogenesis of PDEVs remains incompletely elucidated and diverges from the production pathway of mammalian exosomes. The MISEV2023 guidelines explicitly discourage default use of the term “exosome” in the absence of conclusive biogenesis evidence or validated molecular markers. To advance standardization in future research, we recommend adopting the neutral umbrella term “plant-derived extracellular vesicles (PDEVs)” for general contexts where biogenetic origin is unspecified. When source discrimination is required, naturally secreted vesicles should be designated as PEVs, and heterogeneous mixtures obtained via mechanical disruption should be annotated as PDNVs.
2. Batch-to-batch variations in raw plant materials—including differences in cultivar, growth conditions, and processing methods—have a far greater impact on vesicle composition than the fluctuations typically observed in mammalian cell culture. The lack of standardized raw material specifications and harmonized pre-processing protocols hampers achievement of batch-to-batch consistency. Conventional isolation workflows introduce significant variability in yield, purity, and physicochemical attributes. Even the gold-standard differential ultracentrifugation method yields products that remain contaminated with non-vesicular components. For instance, Koch et al. showed that pellets obtained via differential ultracentrifugation are dominated by cell wall nanofilaments and non-vesicular protein contaminants such as ESM1 and RuBisCO [61]. Moreover, heterogeneity across density gradient fractions is frequently overlooked, with many studies pooling all pellets indiscriminately as “PDEVs” regardless of compositional or biological disparities. For instance, Mammadova et al. demonstrated that low-density fractions were predominantly composed of canonical vesicles, whereas high-density fractions were enriched in virions and debris, exhibiting markedly divergent proteomic profiles [216]. Among emerging isolation modalities, microfluidics enables rapid, efficient, and precise processing of small-volume samples with extremely low reagent consumption. However, disposable-chip costs are prohibitive, and nonspecific lipid adsorption to PDMS and other substrate materials precludes compliance with Good Manufacturing Practice (GMP) mandates for cost control and process robustness. In contrast, TFF supports continuous processing of large-volume feedstocks and eliminates risks of residual sucrose gradient media, representing the leading scalable alternative to conventional ultracentrifugation for GMP-compliant manufacturing. Nevertheless, while size-based sieving alone cannot resolve co-isolating impurities, membrane adsorption compromises yield consistency, particularly when processing high-viscosity plant feedstocks that exacerbate fouling. Therefore, TFF should be positioned in GMP processes as a step for concentrating large-volume samples and performing buffer exchange, and its output still requires further purification through methods such as size exclusion chromatography to meet the purity and batch consistency standards required for PDEVs [214].
3. Currently available markers for PDEVs, such as TET8/TET9, HSP60, HSP70, and GAPDH, have insufficient universality, and PDEVs lack widely recognized surface markers like CD63/CD9/ CD81 found in animal exosomes [82]. The stability of PDEVs varies substantially among plant species. Notably, even at −80 °C, repeated freeze–thaw cycles can induce vesicle fusion and size enlargement. For instance, *Dioscorea* spp. derived nanovesicles remain stable for at least six months at −80 °C, whereas *Rehmannia glutinosa*-derived nanovesicles exhibit severe aggregation and loss of bioactivity after merely two months, regardless of whether stored at −20 °C or −80 °C [129,217]. Furthermore, current single-omics studies face persistent limitations that constrain the reliability and comparability of findings, including the inability of metabolomics and lipidomics to capture rare plant-specific bioactive compounds, coupled with the frequent reliance of transcriptomic annotation of plant miRNAs on generic databases, thereby leading to false-positive identifications. Batch variation in raw materials and isolation process inconsistency are often inadequately corrected, which complicate cross-omics data matching, limit result comparability across studies, and inflate false-positive rates [80].
4. Current mechanistic investigations into PDEVs remain notably superficial, largely confined to the correlational level and lacking causal validation. Most studies merely report alterations in pathway protein expression or perform correlation analyses of microbial abundance, without establishing direct causality through approaches such as receptor knockout, inhibitor intervention, or target gene mutagenesis. For instance, *Andrographis paniculata*-derived PDEVs have been shown to increase the abundance of *Lactobacillus murinus*, yet it remains unclear whether this effect stems from PDEVs directly promoting bacterial growth or indirectly creating a more favorable niche by repairing the intestinal barrier and alleviating inflammation. Existing research frequently attributes the overall bioactivity of PDEVs to a specific miRNA, metabolite, or lipid, but few studies have employed artificial miRNA-knockout or cargo-depleted PDEVs, or conducted component-separation comparisons to verify the independent contribution of each constituent. Moreover, systematic investigation into whether the lipid and protein cargoes of PDEVs participate in cross-kingdom regulation is still lacking. The interaction preferences between PDEVs from different plant sources or cell types and their target cells remain ill-defined. Ginseng-derived PDEVs and citrus-derived PDEVs exert opposing effects on the Firmicutes/Bacteroidetes ratio, yet whether this divergence arises from inherent species-specific traits or from experimental heterogeneity—such as variations in isolation methods or raw material batches—remains unresolved. In addition, the targeting biases and selectivity of PDEVs across different plant origins and cellular contexts have not been systematically compared. Finally, the identification of key phenotypes such as ferroptosis continues to rely on canonical biomarkers; however, these indicators overlap considerably with other forms of programmed cell death, and their specificity and comprehensiveness require further validation.
5. Engineered modification of PDEVs faces a critical technical bottleneck in simultaneously balancing bioactivity preservation, in vivo stability, and targeted delivery capabilities. Regarding drug-loading strategies, while commonly used techniques such as electroporation, sonication, and freeze–thaw cycles can enhance loading efficiency, systematic investigations into their impacts on post-loading vesicular membrane integrity, in vivo behavior, and immunological properties remain scarce. Although surface modifications may reduce immune clearance, certain chemical alterations risk introducing immunogenic epitopes, potentially increasing adverse immune responses. Furthermore, repeated administration could elicit anti-PDEVs antibodies, accelerating vesicle clearance or even triggering hypersensitivity reactions. PDEVs exhibit inherent sensitivity to pH and temperature fluctuations, with hydrogel cross-linking conditions—such as elevated temperatures, acidic environments, and radical-based cross-linkers—readily compromising membrane structural integrity and causing leakage and inactivation of encapsulated bioactive cargoes. Concerning pharmacokinetics and safety, the vast majority of studies only report short-term tissue distribution profiles of PDEVs, leaving key parameters such as systemic half-life, metabolic clearance pathways, and bioavailability inadequately characterized.

Future PDEVs research should focus on standardizing production and regulations, fostering interdisciplinary collaboration, and using advanced technologies to achieve breakthroughs in mechanisms and applications.

1. The ISEV should spearhead the development of unified definitions, nomenclature, and biogenesis characterization protocols for PDEVs to enhance research clarity and cross-study comparability.
2. Priority should be given to establishing plant-specific multi-omics databases that catalogue standard profiles of proteins, nucleic acids, lipids, and metabolites for PDEVs derived from commonly used medicinal plants.
3. Building upon the MISEV2023 framework, there is an urgent need to establish dedicated guidelines for PDEVs research to standardize workflows for isolation, purification, characterization, and quality control. Concurrently, GMP-compliant protocols must be implemented to ensure full traceability and consistency across these processes. To address current technical bottlenecks, future efforts should focus on developing reusable microfluidic chip materials and optimizing TFF membrane compositions and operational parameters to accommodate the unique challenges posed by high-viscosity plant feedstocks.
4. In future investigations, researchers should adopt more rigorous frameworks for mechanistic validation. Establishing causality between PDEVs and observed effects requires employing strategies such as receptor knockout, inhibitor intervention, and target gene mutagenesis. Additionally, conducting component-specific knockdown and overexpression experiments or comparative analyses of fractionated constituents is essential to deconvolute the overall bioactivity of PDEVs and distinguish the individual contributions of specific cargo components.
5. Regarding drug loading, priority should be given to low-shear-force techniques such as low-power pulsed sonication or microfluidic focusing, in combination with membrane stabilizers like trehalose to maximally preserve vesicular integrity. Post-loading EVs should undergo multidimensional characterization, including regarding size, morphology, zeta potential, membrane integrity, and biomarker expression. To minimize immunogenicity, modification strategies should prioritize PEGylation, conjugation with natural ligands, or genetically engineered expression of endogenous modifying proteins. Crucially, comprehensive pharmacokinetic profiling of PDEVs is required, alongside systematic evaluations of long-term toxicity and potential carcinogenic risks to ensure translational safety.

## 6. Limitations

Unlike traditional review articles, our study employs a bibliometric approach to systematically reveal the knowledge structure and research hotspots in the field of PDEVs through objective quantitative data and knowledge mapping. Nevertheless, several limitations of this review should be acknowledged. First, although our search strategy integrated the three core databases (WoSCC, PubMed, and Scopus), the exclusion of specialized databases such as Embase may have resulted in the omission of specific pharmacological or translational studies. Second, due to time constraints, variations in database coverage, and potential self-citation effects, some emerging research hotspots and publications may not have accumulated sufficient citation counts, potentially leading to biased assessments. To ensure data quality, we only included original research articles and reviews, while excluding non-English publications, which may introduce publication and language biases. In addition, the use of different combinations of bibliometric software may result in information loss and slight discrepancies in analytical outcomes. Despite these limitations, our study presents a comprehensive investigation of research hotspots in the biomedical applications of plant exosomes, offering valuable insights for current and future investigations.

## 7. Conclusions

In the past decade, research on PDEVs has swiftly advanced from basic biology to engineered delivery systems. In this review, we employ bibliometric approaches to outline the field’s knowledge structure and offer a detailed overview of its main themes. PDEVs maintain intestinal flora homeostasis by reshaping microbiota and repairing the intestinal barrier while exerting dual regulatory effects on ferroptosis via the NRF2/GPX4/ACSL4 signaling pathway. Diverse loading strategies, surface modification, and membrane fusion techniques have transformed PDEVs from passive natural carriers into smart delivery systems capable of targeted and stimuli-responsive drug release. Therapeutic applications now span beyond intestinal inflammation and include cancer, neurological disorders, tissue regeneration, and metabolic diseases. Despite advancements in the field, several challenges persist, including inconsistent terminology, the absence of standardized isolation protocols for plants, and difficulties related to scalable and reproducible production. Furthermore, the molecular mechanisms underlying cross-kingdom communication and the in vivo fate of PDEVs are still not fully clarified. Future priorities should encompass establishing global nomenclature and plant-specific protocols, developing scalable purification methods, integrating multi-omics approaches with AI for mechanistic discovery, and conducting systematic preclinical safety and pharmacokinetic studies. Addressing these challenges is crucial for the successful translation of PDEVs from research to clinical application.

## Figures and Tables

**Table 1:** Main information about data.

Description	Results
Timespan	2001–2026
Sources	449
Documents	1331
Annual Growth Rate %	40.16
Document Average Age	2.07
Average citations per doc	23.22
References	56,050
**Document Contents**	
Keywords Plus	3331
Author’s Keywords	3259
AUTHORS	
Authors	5877
Authors of single-authored docs	14
**Authors Collaboration**	
Single-authored docs	15
Co-Authors per Doc	8
International co-authorships %	15.23
**Document Types**	
article	1001
review	330

**Table 3:** Extraction and isolation methods for PDEVs.

Method	Principle	Advantages	Limitation	Ref.
Differential Centrifugation	Separates vesicles based on size and density differences using a series of centrifugation steps at increasing speeds.	High purity; simple operation; often considered the “gold standard.”	Time-consuming; requires expensive specialized equipment; may cause vesicle deformation or damage; relatively low yield.	[46,47,48]
Size-Exclusion Chromatography	Separates vesicles based on molecular size differences.	Effectively removes small molecule contaminants; yields high-purity, size-uniform vesicles; preserves vesicle integrity well.	Low yield; high equipment cost; limited sample handling capacity.	[49,50,51]
Polymer Precipitation	Induces vesicle precipitation by reducing solubility using hydrophilic polymers (e.g., PEG) that alter solution properties.	High yield; simple operation; low cost; no special equipment required; suitable for large-scale preliminary enrichment.	Low purity; potential co-precipitation with non-vesicular components, affecting downstream analysis and comparability.	[43,52]
Ultrafiltration	Uses nanoporous membranes with specific pore sizes to retain vesicles larger than the pores while allowing small molecules to pass.	Simple, rapid, economical; does not compromise vesicle bioactivity.	Membrane adsorption or clogging may affect recovery; applied pressure may damage vesicle structure; generally low purity.	[53,54,55]
Tangential Flow Filtration	Based on size exclusion; samples flow tangentially across the membrane surface, retaining particles larger than the pore size while continuously concentrating vesicles.	High purity; high throughput; suitable for large-volume samples; maintains vesicle structural integrity well; scalable.	High equipment cost; relatively complex operation; potential for membrane fouling over prolonged use, requiring regular cleaning.	[56,57,58]
Density Gradient Centrifugation	Samples are placed onto pre-formed density gradients (e.g., sucrose). Under ultracentrifugation, particles settle at positions matching their buoyant density.	High separation efficiency; yields vesicles with very high purity; can distinguish vesicle subpopulations based on density.	Labor-intensive; time-consuming; may cause significant vesicle loss; density medium may co-purify with vesicles, affecting downstream experiments.	[59,60,61,62,63]
Microfluidic	Utilizes physical and chemical property differences in vesicles within microscale channels, achieving rapid separation through specific structures or field effects.	Rapid; high sensitivity; low reagent consumption; low cost.	Currently yields relatively low purity; primarily used for biomarker detection; applications in plant systems are still exploratory.	[64,65,66]
Immunoaffinity Capture	Employs the high specificity of antigen–antibody interactions, using antibodies against specific vesicle surface markers immobilized on magnetic beads or plates to capture target vesicles.	High selectivity; capability of isolating specific vesicle subtypes; high purity; effectively prevents co-isolation of non-vesicular contaminants.	High cost; dependent on known and available specific surface markers; antibody binding may affect vesicle biological function.	[67,68]
Electrophoresis-Dialysis	Under an electric field, charged vesicles migrate towards a specific electrode while selectively retained by a dialysis membrane with defined pore size.	Time-saving; no special equipment required; relatively simple operation.	Limited separation scale; purity may not match that of chromatography or density gradient centrifugation; method adoption requires further validation.	[69,70,71,72]

**Table 5:** Therapeutic effects and mechanisms of PDEVs in inflammatory diseases.

Disease Type	Source	Active Compound	Study Model	Mechanisms	Ref.
Ulcerative colitis	Turmeric	Lipid, protein, and RNA mixture	In vivo: DSS-induced colitis mice In vitro: LPS-stimulated macrophages	Targets inflamed colon, promotes M2 macrophage polarization, modulates gut microbiota, repairs intestinal barrier, suppresses NF-κB pathway.	[156,157]
	*Zanthoxylum bungeanum*	Phenolic acids and alkaloids	In vivo: DSS-induced colitis mice In vitro: LPS-treated THP-1 cells	Reduces colon damage and inflammatory cytokines; repairs intestinal barrier.	[158]
	*Andrographis paniculata*	Lipid mixture	In vivo: DSS/IL-10-/- mice In vitro: NCM460, HCT116, RAW 264.7 cells	Targets inflamed colon. Modulates gut microbiota (enriches L. murinus). Repairs intestinal barrier. Promotes macrophage M2 polarization via IL-4R/PI3K-AKT/JAK-STAT.	[94]
	*Rosa roxburghii*	Lipid and metabolite mixture	In vivo: DSS-induced colitis mice In vitro: RAW 264.7 cells	Rebalances gut microbiota, increase the level of SCFAs, reduces inflammation and oxidative stress, repairs intestinal barrier, modulates Th17/Treg balance.	[159]
	*Prunus mume*	miR159	In vivo: DSS/TNBS-induced colitis mice In vitro: BMDMs, THP-1 cells	Targets inflamed colon; internalized by macrophages; miR159 disrupts NEK7-NLRP3 interaction, inhibiting NLRP3 inflammasome activation.	[160]
	*Taxus chinensis*	Lipid mixture	In vivo: DSS-induced colitis mice In vitro: THP-1 cells	Inhibits TLR4/MyD88/NF-κB pathway, reduces pro-inflammatory cytokines, modulates gut microbiota	[161]
	*Robinia pseudoacacia* L. flower	-	In vivo: DSS-induced colitis mice In vitro: NCM460, MNK3 cells	Activates AhR/IL-22 pathway in ILC3s by modulating gut microbiota-dependent tryptophan metabolism, thereby repairing the intestinal barrier.	[162]
	*Momordica charantia*	Mixture of proteins, RNAs, lipids, metabolites	In vivo: DSS-induced colitis mice In vitro: RAW 264.7 cells	Exerts antioxidant and anti-inflammatory effects; modulates gut microbiota, repairs intestinal barrier.	[163]
	Ginger	Loaded curcumin	In vivo: DSS-induced colitis mice In vitro: LPS-stimulated RAW 264.7 cells	Orally targets colon, reduces inflammation, modulates gut flora, repairs intestinal barrier.	[164]
	Broccoli	Sulforaphane	In vivo: DSS-induced colitis mice In vitro: dendritic cells (DCs)	Activates AMPK in DCs, inducing a tolerogenic phenotype to maintain intestinal immune homeostasis.	[165]
	Rhubarb	Loaded dexamethasone (DXM)	In vivo: DSS-induced colitis mice In vitro: RAW 264.7 cells	Engineered 3-layer nanoplatform enables colon-targeted, reactive oxygen species (ROS)-responsive co-delivery of DXM and REVs; synergistically inhibits NF-κB, scavenges ROS, repairs barrier, and modulates gut microbiota	[154]
	*Houttuynia cordata*	Flavonoids, amino acids, lipids, organic acids	In vivo: DSS-induced colitis mice In vitro: RAW 264.7 cells	Inhibits NLRP3 signaling, modulates gut microbiota, targets inflamed colon, regulates immune cells.	[166]
	Honeysuckle	Mixture of lipids, metabolites, miRNAs	In vivo: DSS-induced colitis mice In vitro: RAW 264.7 cells	Modulates gut microbiota, increases SCFAs, repairs intestinal barrier, rebalances Treg/Th17 response.	[90]
Crohn’s disease	Garlic	Mixture of proteins, RNAs, lipids, metabolites	In vivo: TNBS-induced mice In vitro: TGF-β1-treated fibroblasts	Inhibits PFKFB3 expression, blocks glycolysis, suppresses metabolic reprogramming in fibroblasts to alleviate fibrosis.	[167,168]
Rheumatoid Arthritis	*Pueraria lobata*	gma-miR4412	In vivo: collagen-induced arthritis (CIA) mice; In vitro: *R. gnavus* cultures; Neutrophils	Targets gut bacterium *R. gnavus*; delivers gma-miR4412 to inhibit its PDC expression; reduces PEA production; blocks PEA-BTK-NETs axis to alleviate arthritis.	[152]
	*Morinda officinalis*	Enhanced antioxidant enzymes	In vivo: Adjuvant-induced arthritis mice In vitro: RAW 264.7 macrophages, DC2.4 cells	Inhibits Wnt/β-catenin pathway, promotes macrophage M1-to-M2 polarization; suppresses dendritic cell activation; regulates immune microenvironment	[155]
	Ginger	miR-149 and 6-gingerol	In vivo: CIA mice In vitro: human RA synovial fibroblasts	Suppresses pro-inflammatory cytokines and synovial fibroblast activity components; inhibits KRAS-MAPK pathway.	[169]
	Ginseng	pgi-miR6135j	In vivo: CIA mice In vitro: synovial macrophages	Delivers pgi-miR6135j to synovial macrophages, inhibits KRAS-MAPK pathway	[170]
	Ginger (Folic acid-modified)	6-Gingerol, 6-Shogaol, miRNAs mix	In vivo: CIA mice In vitro: LPS/IFN-γ-stimulated BMDMs	Targets joint M1 macrophages via folate receptor; promotes M2 polarization via PI3K-AKT pathway.	[109]
Osteoarthritis	Ginger	Mixture of identified miRNAs	In vivo: DMM-induced OA rats In vitro: TBHP-induced chondrocytes Ex vivo: Human OA cartilage explants	Attenuates OA progression by activating the Nrf2 pathway, thereby inhibiting oxidative stress and inflammation, and promoting cartilage anabolism	[171]
	Garlic	Mixture of Protein and miRNAs	In vivo: ACLT/DMM-induced OA mice In vitro: IL-1β-treated human chondrocytes	Inhibits phosphorylation of MAPK pathway; upregulates collagen II and aggrecan; downregulates MMP3 and MMP9	[172]
Psoriasis	*Perilla frutescens*	pab-miR396a-5p	In vivo: IMQ-induced psoriasis mice In vitro: IL-6-stimulated HaCaT cells	Targets HSP83a/HSP90 to suppress NF-κB and JAK-STAT signaling, inhibiting IL-17 pathway; incorporated into hydrogel for enhanced transdermal delivery	[76]
	Rose Hip	β-carotene and gallic acid	In vivo: IMQ-induced psoriasis mice In vitro: HaCaT keratinocytes; RAW264.7 macrophages	Scavenges ROS; inhibits epidermal hyperplasia and macrophage infiltration; reduces inflammatory cytokines	[173]
	Garlic, Shallot	-	In vivo: IMQ-induced psoriasis mice In vitro: HaCaT keratinocytes; RAW264.7 macrophages	Promotes keratinocyte differentiation and exerts anti-inflammatory effects; activates the NRF2 antioxidant pathway to suppress upstream IL-17-driven inflammation.	[174]
Periodontitis	*Paris polyphylla var. yunnanensis* leaf	-	In vitro: Human PDLSCs, P. gingivalis LPS stimulation In vivo: SD rat alveolar bone defect model	Enhances PDLSC proliferation, migration, osteogenic differentiation. Suppresses IL-6/IL-8. Promotes bone regeneration. Modulates macrophage M1→M2 polarization.	[175]
	Garlic	-	In vivo: Ligature-induced periodontitis mice In vitro: LPS-stimulated human gingival fibroblasts	Upregulates PHGDH, activates the PI3K/AKT pathway, enhances Nrf2/mTOR, inhibits NF-κB, reduces inflammation/oxidative stress, and improves mitochondrial function.	[176]
	*Scutellaria baicalensis*	Baicalin, Baicalein, Wogonoside, Wogonin	In vitro: Human PDLSCs stimulated with Pg-LPS and TNF-α	Possesses anti-P. gingivalisactivity and TNF-α-neutralizing capacity. Alleviates inflammation and promotes osteogenic differentiation of PDLSCs partly via delivering miR-21-5p to inhibit the STAT3 pathway.	[177]
	Ginger	-	In vivo: Ligature-induced periodontitis in SD rats. In vitro: LPS-stimulated human periodontal ligament fibroblasts	Promotes PDLF proliferation and migration; reduces intracellular ROS by inhibiting the NF-κB signaling pathway; alleviates oxidative stress and inflammation.	[178]
	Ginseng	Ginsenosides (Rg1, Rg3, Rb1, Re)	In vivo: Ligature-induced periodontitis in SD rats In vitro: LPS-stimulated RAW264.7 & H2O2-stimulated MC3T3 cells	Exhibits antibacterial and anti-biofilm activity; promotes macrophage M2 polarization, reduces pro-inflammatory cytokines, and scavenges ROS; enhances osteogenic and angiogenic differentiation	[116]
	*Curcuma kwangsiensis*	Curcuminoids	In vitro: Human PDLSCs, HUVECs, RAW264.7 macrophages, hFOB 1.19 cells	Promotes PDLSC proliferation/migration; Induces macrophage M2 polarization; Enhances angiogenesis and osteogenic differentiation.	[179]

## Data Availability

No new data were created or analyzed in this study. Data sharing is not applicable to this article.

## Supplementary Materials

The following supporting information can be downloaded at https://www.mdpi.com/article/10.3390/ph19081156/s1, Supplementary Table S1. Literature Retrieval Strategy. Supplementary Table S2. Co-citation network information. Supplementary Table S3. Statistical validation of keyword co-occurrence clusters against null models. Supplementary Table S4. Co-occurrence network information.

## Abbreviations

The following abbreviations are used in this manuscript:

PDEVs	Plant-derived extracellular vesicles
EVs	Extracellular vesicles
MVBs	Multi-vesicular bodies
WoSCC	Web of Science Core Collection
DOI	Digital Object Identifier
TLS	Total Link Strength
ISEV	The International Society for Extracellular Vesicles
VIB	vesicle isolation buffer
AWF	apoplastic wash fluid
DXM	dexamethasone
RNA-seq	RNA sequencing
CL-EVs	Chinese leek-derived extracellular vesicles
GELNs	Garlic-derived exosome-like nanoparticles
CELNs	citrus exosome-like nanoparticles
SCFAs	Short-chain fatty acids
HNVs	Honeysuckle-derived nanovesicles
GENs	Ginseng-derived exosome-like nanoparticles
MC-EVLPs	Momordica charantia-derived extracellular vesicle-like particles
HT-EVLPs	Houttuynia cordata Thunb-derived extracellular vesicle-like particles
HELNs	Huangqi-derived exosome-like nanoparticles
PLDENs	Pueraria lobata-derived exosome-like nanovesicles
APN	anti-periostin peptide
LEVs	Lemon extracellular vesicles
PDRN	polydeoxyribonucleotide
DOX	doxorubicin
ROS	reactive oxygen species
CAEs	Centella Asiatica-derived exosomes
PEA	phenylethylamine
REVs	rhubarb-derived extracellular vesicles
MOE	Morinda officinalis-derived nanovesicles
DCs	dendritic cells
CIA	collagen-induced arthritis
GExos	ginseng-derived exosomes
SMEVs	Salvia miltiorrhiza-derived extracellular vesicles
PMB	polymyxin B
LELNs	lavender-derived exosome-like nanoparticles
PNVs	Panax notoginseng-derived nanovesicles
GPEVs	Gynostemma pentaphyllum-derived extracellular vesicles
PMENs	Polygonum multiflorum-derived exosome-like nanovesicles
BBB	Blood–brain barrier
PDA	polydopamine
PDNs	Panax notoginseng-derived exosome-like nanoparticles
CS-rLNPs	Celery seed-derived reconstituted lipid nanoparticles
PEVs	plant extracellular vesicles
PDNVs	plant-derived nanovesicles
GMP	Good Manufacturing Practice
